# Binding Modes of Peptidomimetics Designed to Inhibit STAT3

**DOI:** 10.1371/journal.pone.0051603

**Published:** 2012-12-12

**Authors:** Ankur Dhanik, John S. McMurray, Lydia E. Kavraki

**Affiliations:** 1 Department of Computer Science, Rice University, Houston, Texas, United States of America; 2 Department of Experimental Therapeutics, University of Texas MD Anderson Cancer Center, Houston, Texas, United States of America; University of Akron, United States of America

## Abstract

STAT3 is a transcription factor that has been found to be constitutively activated in a number of human cancers. Dimerization of STAT3 via its SH2 domain and the subsequent translocation of the dimer to the nucleus leads to transcription of anti-apoptotic genes. Prevention of the dimerization is thus an attractive strategy for inhibiting the activity of STAT3. Phosphotyrosine-based peptidomimetic inhibitors, which mimic pTyr-Xaa-Yaa-Gln motif and have strong to weak binding affinities, have been previously investigated. It is well-known that structures of protein-inhibitor complexes are important for understanding the binding interactions and designing stronger inhibitors. Experimental structures of inhibitors bound to the SH2 domain of STAT3 are, however, unavailable. In this paper we describe a computational study that combined molecular docking and molecular dynamics to model structures of 12 peptidomimetic inhibitors bound to the SH2 domain of STAT3. A detailed analysis of the modeled structures was performed to evaluate the characteristics of the binding interactions. We also estimated the binding affinities of the inhibitors by combining MMPB/GBSA-based energies and entropic cost of binding. The estimated affinities correlate strongly with the experimentally obtained affinities. Modeling results show binding modes that are consistent with limited previous modeling studies on binding interactions involving the SH2 domain and phosphotyrosine(pTyr)-based inhibitors. We also discovered a stable novel binding mode that involves deformation of two loops of the SH2 domain that subsequently bury the C-terminal end of one of the stronger inhibitors. The novel binding mode could prove useful for developing more potent inhibitors aimed at preventing dimerization of cancer target protein STAT3.

## Introduction

**Figure 1:**
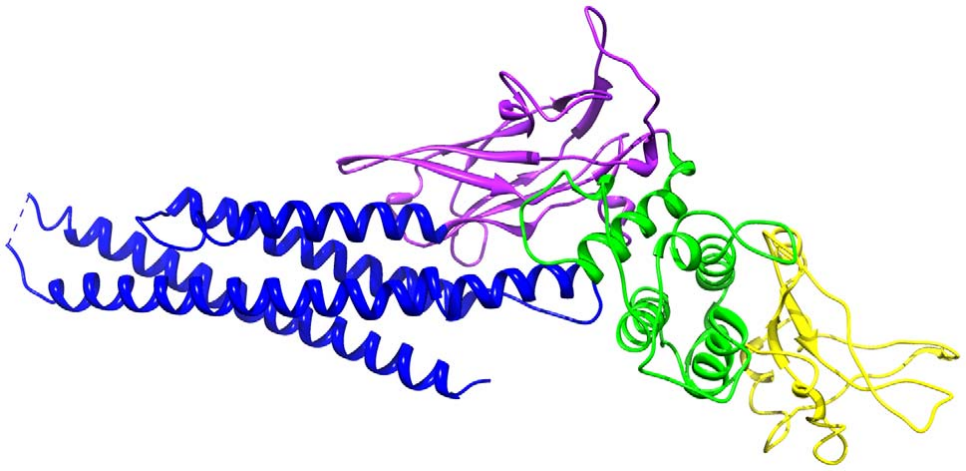
STAT3 structure. Protein Data Bank (ID 1BG1) structure of STAT3 is shown. The structure has four domains: a N-terminal four-helix bundle (in blue, residues 138–320), an eight-stranded 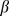-barrel (in purple, residues 321–465), an 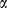-helical connector domain (in green, residues 466–585), and a SH2 domain (in yellow, residues 586 to 688).

Development of effective therapeutics is the ultimate goal of cancer research [1]–[6], but it is a time-consuming and expensive process [7]–[10]. Structure-based computational techniques [11], [12] such as virtual screening [13]–[15], docking [16], [17], and molecular dynamics [18], [19] have proven useful in the development of drugs. Even if there have not been many successful drug discovery stories based on computation alone, the use of structure-based computational techniques has helped gain better understanding of how a putative drug compound binds to its target receptor, and has reduced the drug development time and costs [20]–[22]. In this paper, we discuss computational modeling of binding interactions between a specific set of peptidomimetic inhibitors [23]–[26] and the Src-homology 2 (SH2) domain of STAT3 or Signal Transducer and Activator of Transcription 3 [27] (Figure 1). STAT3 is constitutively activated in a number of human cancer types such as lung cancer, breast cancer, multiple myeloma, and others [28]–[30]. The Jak-STAT pathway [31], [32] describes the mechanism of action that leads to the transcription of anti-apoptotic genes. Upon extracellular signaling, a series of phosphorylations of cell surface receptors and Janus kinases (JAKs) inside the cell results in the phosphorylation of STAT3. A phosphorylated STAT3 then forms a dimer via its SH2 domain and the dimer translocates to the nucleus where it is involved in the transcription process.

Our focus in this work is on 12 peptidomimetic [23]–[26](mimic pTyr-Xaa-Yaa-Gln motif) inhibitors that target the SH2 domain of STAT3 with the aim of preventing the dimerization of STAT3, and subsequent translocation and transcription. The experimental structures of the peptidomimetics bound to the SH2 domain are unavailable. However, the experimental binding affinities, which measure the thermodynamic stability of binding interactions between the peptidomimetics and the SH2 domain, have been derived using fluorescence polarization [33]. Our goal is to computationally model the binding modes which define how a conformation of a peptidomimetic binds to the conformation of the SH2 domain, analyze the binding interactions, estimate the binding affinities, and calculate the correlation between the estimated and the experimental binding affinities.

Our computational modeling approach combines molecular docking and molecular dynamics and derives inspiration from previous work [18], [24], [34]–[39]. Given a protein and an unbound ligand, molecular docking computes the preferred conformation and location of the ligand in the binding pocket of the protein. Many molecular docking programs exist (see representative examples [40]–[48]) and several docking studies have been performed with varied amount of success (e.g., [49]–[57]). Three major limitations however remain.

- A docking program typically computes the best conformation and placement of the ligand such that it minimizes an energy function specific to the docking program. The energy function approximates the free energy of binding and, in general, accuracy of the binding energy is sacrificed so that the computation of energy can be performed in minimal time. The approximate energy functions, therefore, may result in conformations that are not accurate [58]–[60].
- Most docking programs treat the protein as a rigid molecule or, at the very best, a molecule with limited flexibility. Thus, most of these programs perform what is known as flexible ligand docking to a rigid receptor. However, it is well known that more accurate modeling of binding interactions between a ligand and a receptor requires accounting for the flexibility of the receptor [61], [62].
- Docking of small ligands with 5 or 6 rotatable bonds is fairly accurate and computationally fast. However, docking of large ligands with many rotatable bonds, such as the peptidomimetic inhibitors in our dataset, is inaccurate and computationally expensive. A large number of rotatable bonds increases the dimensionality of the conformation space of the ligand which makes searching for the docked conformation extremely challenging and time-consuming [57], [63], [64].

Our modeling approach addresses the above limitations in the following way. Docking of a peptidomimetic is first done with an AutoDock [44], [65]-based incremental docking protocol that we have developed recently [66]. A molecular dynamics simulation of the docked conformation of the peptidomimetic in complex with the SH2 domain is then performed. Using molecular dynamics, we are able to treat both the ligand and the receptor as flexible and, more importantly, we analyze deformations in the structure of the complex in a simulated solvent environment. The physics-based force field used in the molecular dynamics simulations is more detailed and accurate as compared to the energy functions used in molecular docking. Molecular dynamics simulation thus also lends itself to calculation of more accurate binding affinity estimates [67], [68].

Using our modeling approach, we show that we were able to obtain various binding modes for the peptidomimetics. Not only did we obtain previously proposed binding modes [24], [69], but we also obtained a novel binding mode. The estimated binding affinities and the experimental binding affinities are well correlated which validates our modeling approach. By using the estimated binding affinities and conformational analysis of the molecular dynamics trajectories, we are able to differentiate between strong and weak binders. In the following section, we present details of our peptidomimetic dataset, explain our computational modeling approach, binding affinity calculations, and data analysis techniques. This is followed by a description of the results from the computational modeling of the peptidomimetics in complex with the SH2 domain. Finally we conclude with an overall discussion of our work.

## Methods

### Dataset

The 12 inhibitors used in this study were obtained from a series of 142 peptidomimetic compounds [23]–[26]. These 142 peptidomimetics mimic pTyr-Xaa-Yaa-Gln motif and were developed to bind to the SH2 domain with the purpose of inhibiting the activity of STAT3. The binding affinities (measured as IC_50_ values) of the 142 peptidomimetics were evaluated using fluorescence polarization [33]. The IC_50_ value gives the concentration of the peptidomimetic that is required to competitively inhibit the binding of FAM-Ala-pTyr-Leu-Pro-Gln-Thr-Val-NH_2_ (FAM = 5-carboxyfluorescein) to Stat3 by 50% [23]. The binding affinities of the 142 peptidomimetics were found to range from weak (IC_50_ = 100,000 nM) to strong (IC_50_ = 39 nM).

**Figure 2:**
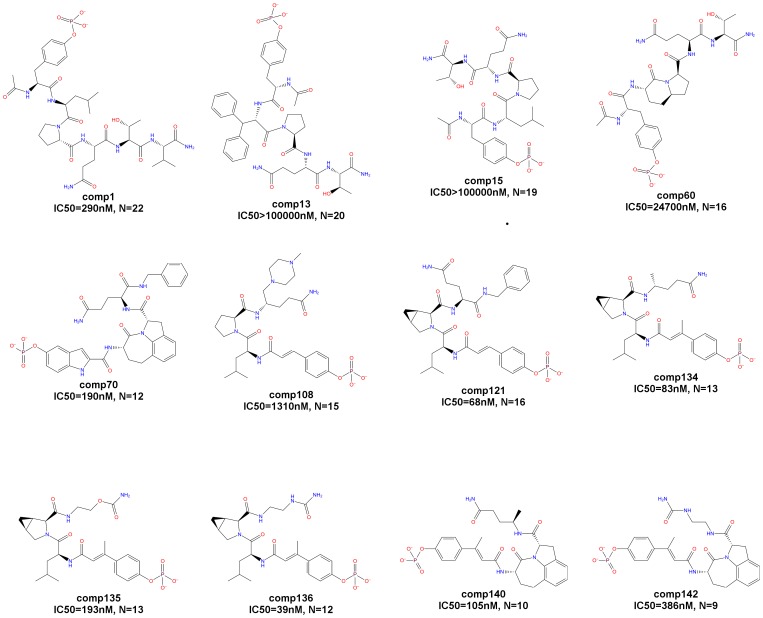
12 peptidomimetics. 2-D chemical representations of the 12 peptidomimetics that form our dataset are shown. IC_50_ value represents the experimental binding affinity of each peptidomimetic derived using fluorescence polarization and N represents the number of rotatable bonds in each peptidomimetic.

The molecular dynamics simulation, which is part of our modeling approach, and the method used for the estimation of binding affinities (see sections below) are computationally expensive. Therefore, we limited our modeling study to 12 peptidomimetics. The 12 peptidomimetics used in this study were chosen such that they represent a range of values of the experimental binding affinities as shown in Figure 2 and also represent a range of sizes varying from 9 torsional degrees of freedom to 22 torsional degrees of freedom. Each peptidomimetic was named such that the compound number represents the order in which the peptidomimetic appears in the original publications [23]–[26] where the 142 peptidomimetics were first described.

The structure of STAT3 was obtained from the Protein Data Bank [70] (PDB ID 1BG1). The structure contains residues 136 to 716 of Stat3, half a DNA duplex, and 127 water molecules per asymmetric unit [27]. Since we are interested in the modeling of the peptidomimetics bound to the SH2 domain, the structure of the SH2 domain corresponding to residues 585 to 688 (Figure 1) was isolated. The water molecules and the DNA duplex were ignored. Using the molecular builder of the Maestro software [71] (version 9.1), the 2-D chemical representations of the 12 peptidomimetics (Figure 2) were converted to 3-D structures of the unbound peptidomimetics.

### Modeling Approach

Our two-step computational modeling approach combined molecular docking and molecular dynamics. Molecular docking of a large ligand such as a peptidomimetic with many rotatable bonds is challenging. A large ligand spans a high-dimensional conformation space which makes exploration of docked conformation of the ligand challenging. Our recently developed Autodock-based incremental docking protocol has been shown to improve docking of large ligands [66]. Therefore, we first docked the 12 peptidomimetic inhibitors in our dataset to the SH2 domain of STAT3 using our incremental docking protocol [66], and subsequently performed molecular dynamics simulations of the docked conformations of the peptidomimetics in complex with the SH2 domain.

**Figure 3:**
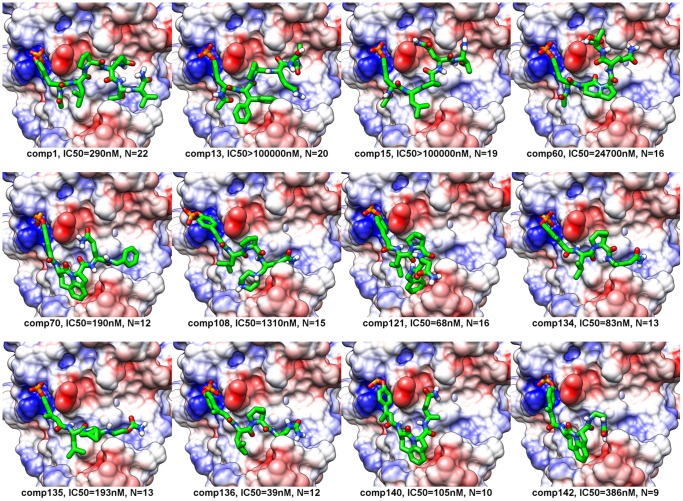
Docked conformations. Docked conformation of each peptidomimetic that was obtained using our incremental docking protocol is shown. The peptidomimetic conformation (in green) is shown in complex with the SH2 domain of STAT3 (in surface representation). The surface coloring shows the Coulombic electrostatic potential in different regions of the surface of the SH2 domain. The potential ranges from positive (in blue) to negative (in red). IC_50_ value represents the experimental binding affinity of each peptidomimetic derived using fluorescence polarization and N represents the number of rotatable bonds in each peptidomimetic.

**Figure 4:**
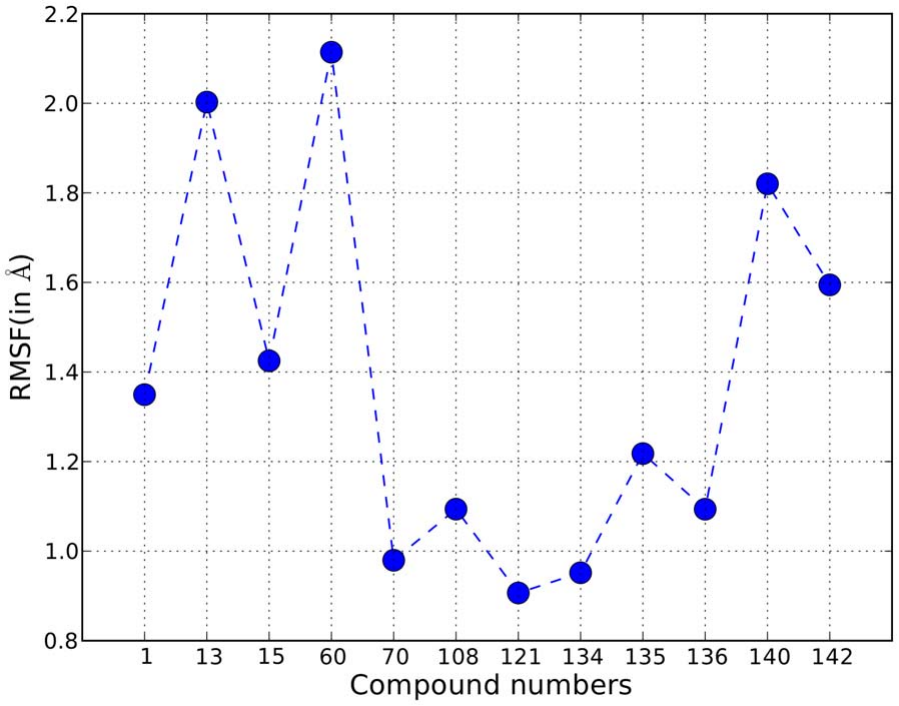
Root mean square fluctuations. Root mean square fluctuation (RMSF) of the 12 peptidomimetics in complex with the SH2 domain of STAT3 is shown. Each RMSF value was computed using 1000 conformations of the peptidomimetic derived from the 10 ns molecular dynamics trajectory.

Starting from a fragment of the ligand, at each incremental step, our docking protocol explores a few rotatable bonds, then selects a small number of best partially docked fragments, grows the fragments by adding few more rotatable bonds and atoms, and docks again. The dock-select-grow-dock process is repeated until all the rotatable bonds in the ligand are explored. AutoDock [44], [65] is used in each step to explore only a few rotatable bonds and this makes the docking operation fast and accurate.

Each peptidomimetic in our dataset was docked to the SH2 domain of STAT3 using our incremental docking protocol. Since the phosphate group of the pTyr residue in each peptidomimetic is known to bind to the sub-pocket formed by residues Lys591, Arg609, Ser611, Glu612, and Ser613 [24], [69], at each incremental docking step we selected conformations with the lowest values of scoring function *S*, where
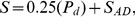
(1)

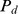
 is the squared distance of the phosphorus atom (in pTyr) from coordinates 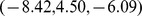 that represent approximate center of the sub-pocket, and 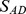 is the binding affinity estimated by AutoDock’s energy function. The scoring function S, thus, penalizes large distance between the phosphate group and the sub-pocket. The details of the incremental docking of peptidomimetics and scoring function S are available in the Supporting Information (Section S1).

**Figure 5:**
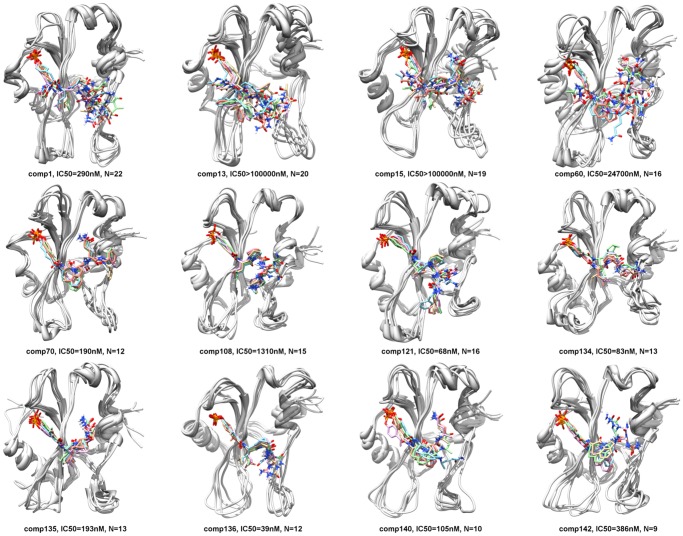
Representative conformations after clustering. For each peptidomimetic, 5 conformations in complex with the SH2 domain (in gray) of STAT3 are shown. The 5 conformations are the representatives of the 5 clusters obtained after k-means clustering of the 1000 conformations that were derived from 10 ns molecular dynamics trajectory of each peptidomimetic-SH2 domain complex. IC_50_ value represents the experimental binding affinity of each peptidomimetic derived using fluorescence polarization and N represents the number of rotatable bonds in each peptidomimetic.

After all the rotatable bonds in a peptidomimetic were explored and all its atoms were docked, the docked conformation of the peptidomimetic with the lowest value of *S* (see, equation (1)) was selected. For each peptidomimetic in our dataset, molecular dynamics simulation of the selected docked conformation, in complex with the SH2 domain of STAT3, was performed. The *sander* module in the AMBER11 software package [72] was used for the simulation. The peptidomimetic inhibitor was described with generalized amber force field [73] (GAFF), and point charges were calculated for the atoms using *antechamber* module (from AmberTools software package [74] version 1.5) and AM1-BCC charge model. The protein was described with AMBER’s ff99SB force field. The complex was solvated in a 15 Å octahedral box of TIP3P water and the whole system was neutralized by adding Na^+^ counterions according to the net charge of the peptidomimetic. Table S1 lists the number of atoms in each of the 12 molecular dynamics systems.

**Figure 6:**
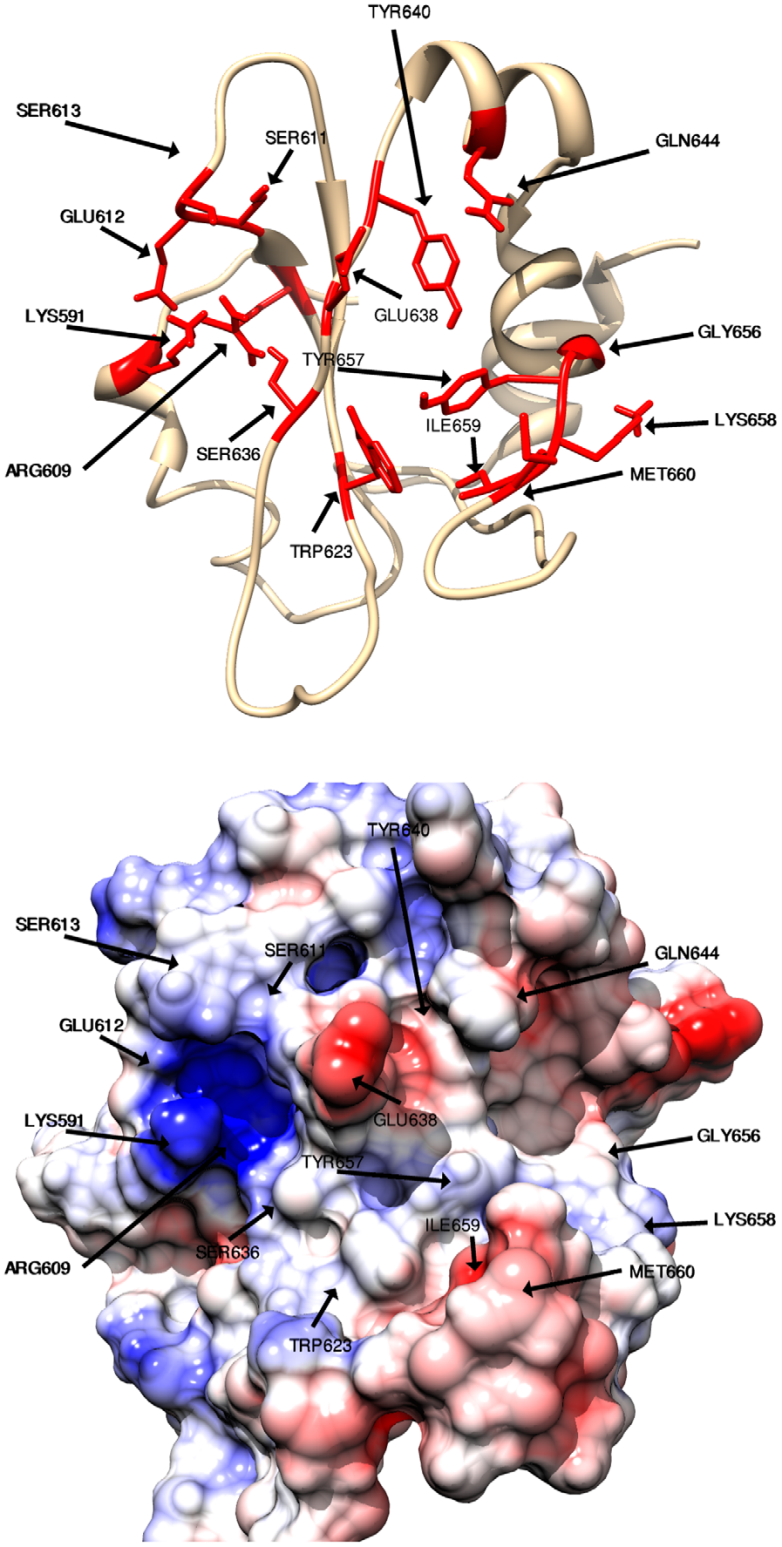
Residues involved in hydrogen bonds. The residues (labeled) of the SH2 domain that form hydrogen bonds with at least one of the 12 peptidomimetics are shown. The top figure shows a cartoon representation of the SH2 domain and the bottom figure shows a surface representation. The surface coloring shows the Coulombic electrostatic potential in different regions of the surface of the SH2 domain. The potential ranges from positive (in blue) to negative (in red). Note that a hydrogen bond is ignored if it is present in less than 50% of the conformations in the 10 ns molecular dynamics trajectory.

The complex was first minimized using 100 cycles of steepest descent minimization followed by 1900 cycles of conjugate gradient minimization. This was followed by 50 ps of temperature equilibration where the temperature was raised from 100 K to 300 K using Berendsen [75] control with coupling parameter set to 2 ps. Pressure equilibration was then performed for 200 ps using Berendsen control with pressure relaxation time set to 2 ps. Finally, a production simulation of 10 ns was performed at constant temperature and pressure, and the trajectory was output at every 10 ps. During the molecular dynamics simulation, SHAKE algorithm was used to constrain bonds involving hydrogen atoms and therefore forces for the bonds involving hydrogen atoms were not calculated. For computing electrostatic energies, Particle Mesh Ewald [76] (PME) method was used with the non-bonded cutoff set to the default value of 8 Å. Plots showing variation of system properties (total energy, potential energy, temperature, and pressure) during the production simulation are available in the Supporting Information (Figures S1, S2, S3, S4, S5, S6, S7, S8, S9, S10, S11, and S12) and reveal equilibrated and stable systems.

**Figure 7:**
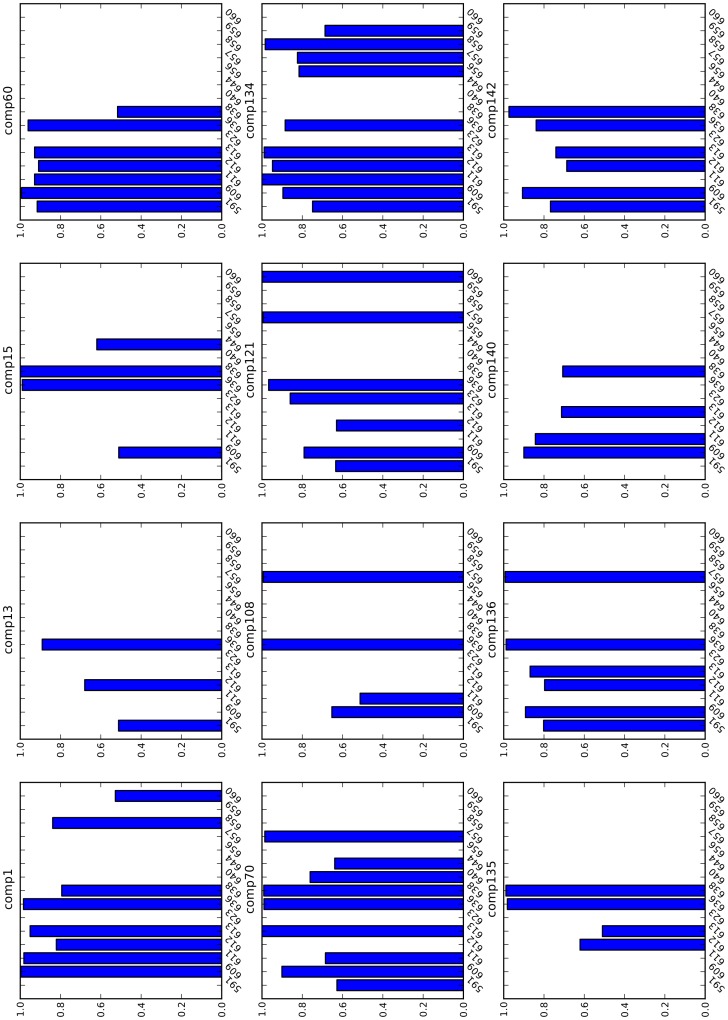
Hydrogen bond occupancy. Hydrogen bond occupancy plots for each peptidomimetic are shown. In each sub-plot, the x-axis represents the serial numbers of the residues of the SH2 domain of STAT3 and the y-axis represents the hydrogen bond occupancy value for a given residue. Hydrogen bond occupancy is computed as the fraction of conformations out of 1000 conformations of a peptidomimetic in which the given residue participates in a hydrogen bond. The 1000 conformations of each peptidomimetic were derived from the corresponding 10 ns molecular dynamics trajectory. Note that a hydrogen bond is ignored if it is present in less than 500 conformations.

To evaluate the accuracy of our modeling approach, we performed a study where we compared the structures modeled using our approach with experimentally-derived structures. Since experimentally determined structures of the peptidomimetics in complex with the SH2 domain of STAT3 or any other protein from the STAT family are unavailable, the validation was done using a dataset of similar complexes derived from the PDBbind database [77]. The details and analysis of the validation study are available in the Supporting Information (Section S2). The analysis (Figures S13, S14, S15, S16, S17, S18, S19, S20, S21, and S22, Table S2) shows that the modeled structures and experimental structures are spatially close and therefore we conclude that our modeling approach is well-suited for modeling of peptidomimetic-SH2 complexes that are described in this paper.

### Binding Affinities

Trajectories obtained from molecular dynamics simulations were also used to estimate binding affinities. The binding affinity of each peptidomimetic in complex with the SH2 domain was obtained using Molecular Mechanics Poisson Boltzmann (or Generalized Born) Surface Area (MMPB/GBSA) calculations [78]. The binding affinity is given by
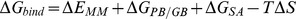
(2)where, *T* is the temperature, 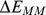 represents the molecular mechanics energy, 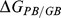 represents the polar part of solvation energy, 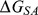 represents the non-polar part of the solvation energy, and 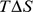 represents the energetic penalty due to loss of entropy upon binding.

**Figure 8:**
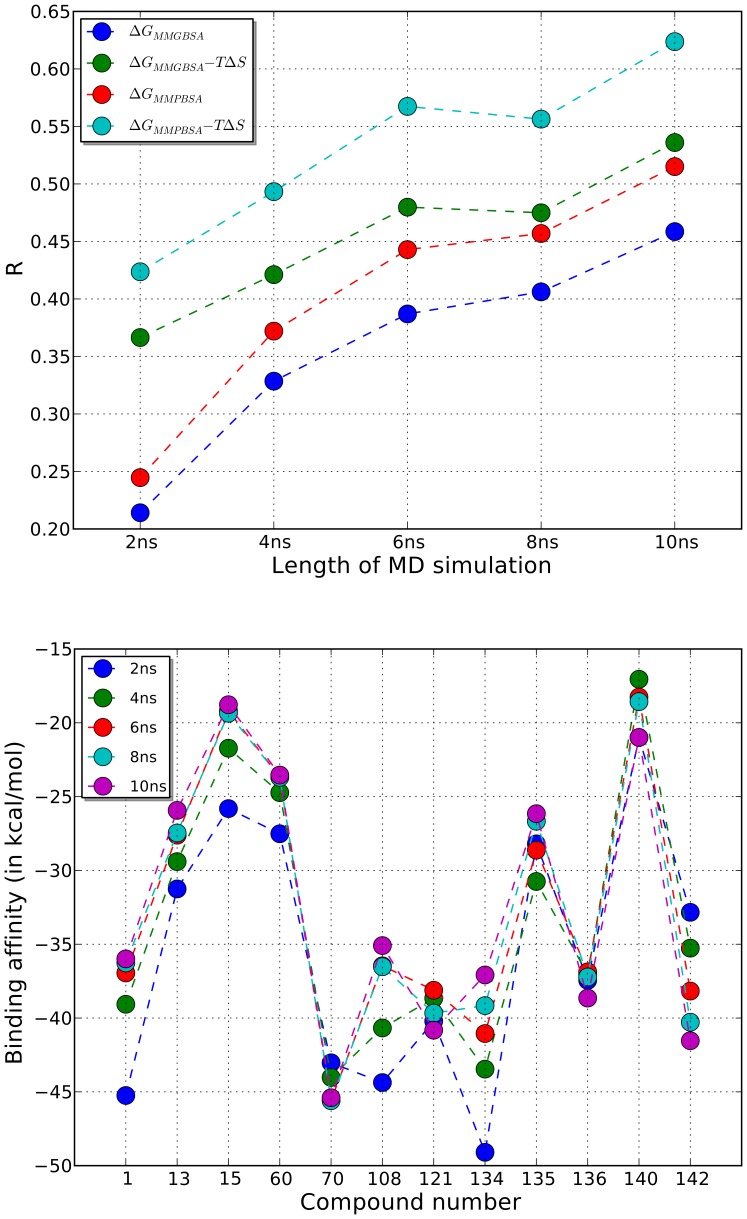
Correlation between experimental and esimated affinities. The top figure shows the variation of the Pearson correlation coefficient (R), computed between the experimental binding affinities and the estimated binding affinities of the 12 peptidomimetics, with the length of molecular dynamics simulation. The binding affinities were estimated using 4 different schemes. 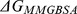 and 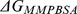 represent non-entropic contribution to the binding affinity computed using the MMGBSA and MMPBSA methods in AmberTools software package. 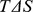 represents the entropic contribution computed using the *nmode* method in AmberTools. The bottom figure shows, for each peptidomimetic, the estimated binding affinity value computed using 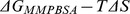 scheme. Because the values computed using MMGBSA, MMPBSA, and nmode methods are averaged over the snapshots of the molecular dynamics trajectory, we also plot the variation of estimated binding affinity values with increasing length of the molecular dynamics simulation.

To compute 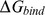, values of 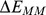, 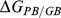, and 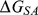 were computed averaged over the snapshots of the molecular dynamics production trajectory. The 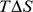 value was computed from a normal mode analysis of the system using 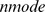 module of the AmberTools [74] package. For entropy computation, every 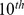 snapshot of the molecular dynamics trajectory was used. Calculations of all of the above 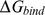 components were done using MMPBSA script provided by the AmberTools package. For computing the polar part of solvation energy using Poisson-Boltzmann calculations, the ionic strength was set to 0.1 mM. All other parameters needed by MMPBSA script were kept at their default values.

### Data Analysis

Trajectory data obtained after the 10 ns molecular dynamics simulation was analyzed in a variety of ways. Prior to analyzing the data, however, the following processing was done. Water and counterions were removed from the trajectory data. All atoms were moved such that the center of mass of the complex moved to the center of the simulation box and imaging was done to bring all atoms inside the primary unit cell. All conformations of the complex contained in the snapshots of the trajectory were then fitted to the conformation in the first frame of the production simulation. Mass-weighted root mean squared distance (RMSD) fitting was done. Note that since we output trajectory at every 10 ps, we obtained 1000 snapshots or conformations of the complex from a 10 ns molecular dynamics simulation.

**Figure 9:**
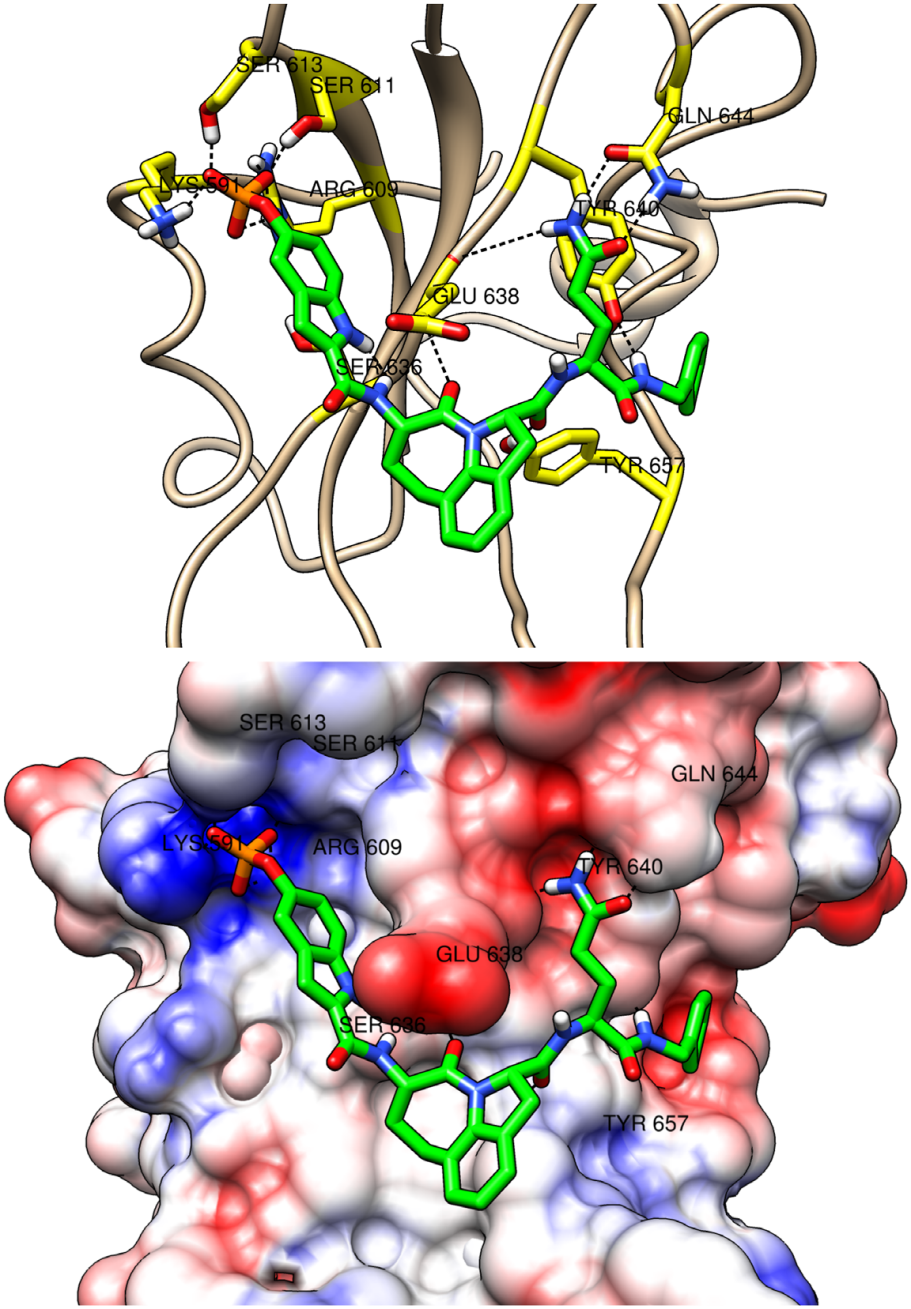
Bent binding mode. The bent binding mode of peptidomimetic comp70 (in green) is shown. The peptidomimetic is in complex with the SH2 domain of STAT3 which is shown in cartoon (top) and surface (bottom) representations. The residues of the SH2 domain which participate in hydrogen bonds are labeled. The top figure also shows the hydrogen bonds (dashed lines) that the residues (in yellow) participate in. The surface coloring shows the Coulombic electrostatic potential in the different regions of the surface of the SH2 domain. The potential ranges from positive (in blue) to negative (in red). The IC_50_ value for comp70 is 190 nM.

**Figure 10:**
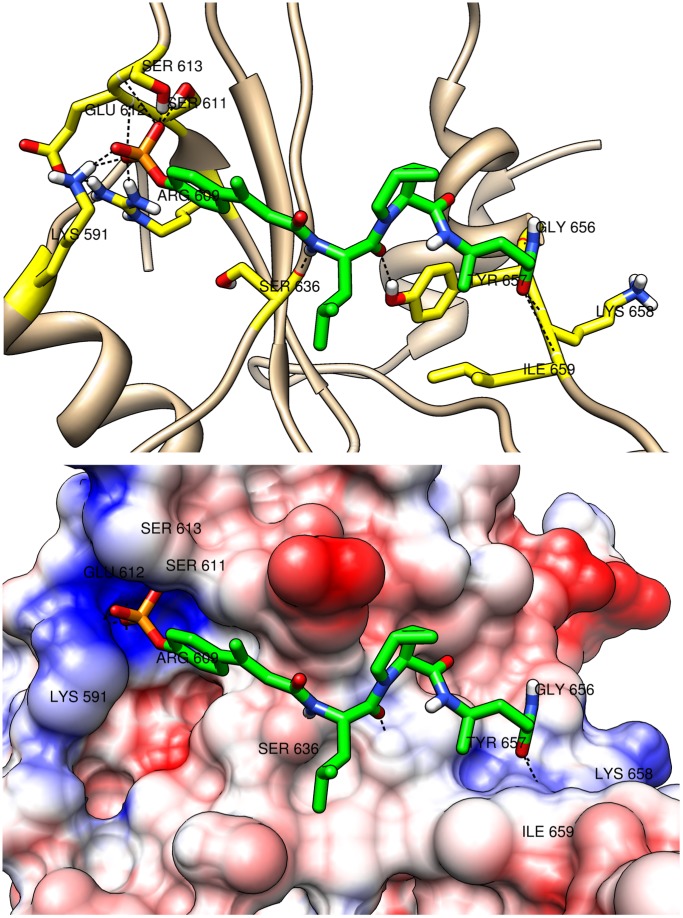
Extended binding mode. The extended binding mode of peptidomimetic comp134 (in green) is shown. The peptidomimetic is in complex with the SH2 domain of STAT3 which is shown in cartoon (top) and surface (bottom) representations. The residues of the SH2 domain which participate in hydrogen bonds are labeled. The top figure also shows the hydrogen bonds (dashed lines) that the residues (in yellow) participate in. The surface coloring shows the Coulombic electrostatic potential in the different regions of the surface of the SH2 domain. The potential ranges from positive (in blue) to negative (in red). The IC_50_ value for comp134 is 83 nM.

**Figure 11:**
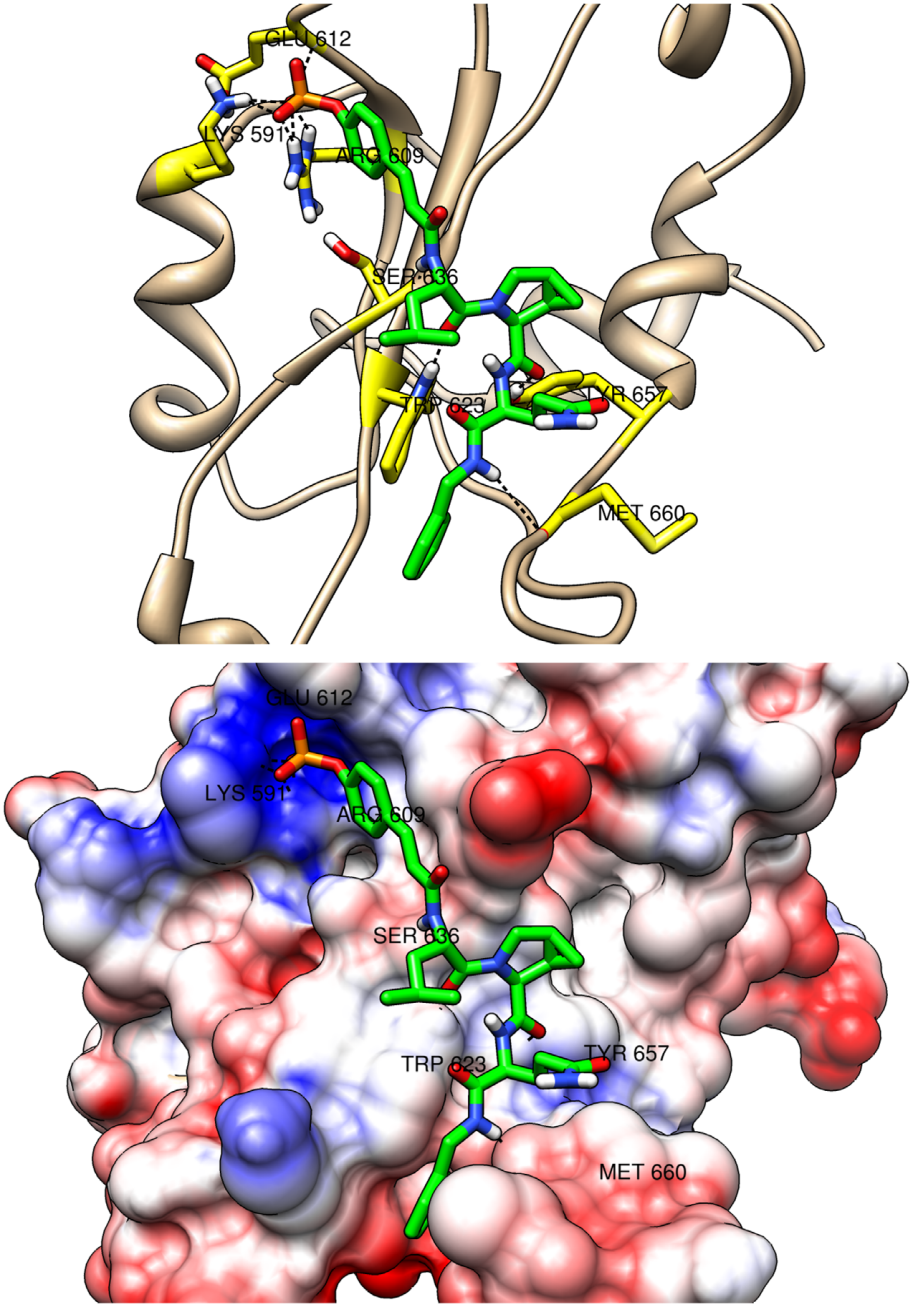
Wedged binding mode. The proposed novel wedged binding mode of peptidomimetic comp121 (in green) is shown. The peptidomimetic is in complex with the SH2 domain of STAT3 which is shown in cartoon (top) and surface (bottom) representations. The residues of the SH2 domain which participate in hydrogen bonds are labeled. The top figure also shows the hydrogen bonds (dashed lines) that the residues (in yellow) participate in. The surface coloring shows the Coulombic electrostatic potential in the different regions of the surface of the SH2 domain. The potential ranges from positive (in blue) to negative (in red). The IC_50_ value for comp121 is 68 nM.

Average (mass-weighted) root mean square fluctuations (RMSF) were computed for each peptidomimetic bound to the SH2 domain. The RMSF value represents the average value of the RMSD between the peptidomimetic conformation in the first frame of the molecular dynamics trajectory and the conformations in the subsequent frames. Thus, the RMSF value is indicative of the time-averaged fluctuation of the peptidomimetic conformation. Clustering of conformations of the peptidomimetic was done and conformations that are representative of the clusters were identified. Clustering was done using k-means [79] (k was set to 5) algorithm with RMSD as the similarity metric. Hydrogen bonds are critical for stabilizing the binding interactions [80]–[83] and were identified between each peptidomimetic and the SH2 domain. If a hydrogen bond was present in less than 50% of the conformations in the trajectory, it was ignored. For each peptidomimetic in complex with the SH2 domain, we computed the hydrogen bond occupancy of the residues of the SH2 domain. Hydrogen bond occupancy of a residue is defined as the fraction of conformations in the molecular dynamics trajectory that contain at least one hydrogen bond involving that particular residue. Computation of RMSF values and k-means clustering was done using *ptraj* module from the AmberTools [74] package. Hydrogen bonds were identified using *hbond* tool in the Chimera software package [84] version 1.6.

## Results

### Conformational Analysis

Figure 3 shows the best docked conformation, of each of the 12 peptidomimetics, computed using the incremental docking protocol. These docked conformations were then solvated and subjected to 10 ns of molecular dynamics simulations. Snapshots of the trajectories were output at every 10 ps and therefore we obtained 1000 conformations for each of the 12 pepetidomimetic-SH2 domain complexes. The RMSF value for each peptidomimetic is shown in Figure 4. The RMSF value quantifies the average spatial fluctuation of the peptidomimetic conformation in the 1000 snapshots. A low RMSF value is thus indicative of spatial stability of the conformation of the peptidomimetic bound to the SH2 domain. The RMSF values for weak binders such as comp13, comp15, and comp60 are higher as compared to the RMSF values of the strong binders such as comp70, comp121, comp134, comp135, and comp136. As an exception, comp140, another strong binder, shows surprisingly large RMSF value (1.82 Å) that is comparable to the RMSF values of the weak affinity peptidomimetics.

Through clustering of the 1000 conformations, we obtained 5 representative conformations of each peptidomimetic-SH2 complex (Figure 5). All representative conformations have the phosphate group of the pTyr residue or its surrogate in the location of the corresponding pTyr705 in the crystal structure of STAT3 [27]. The representative conformations of the strong binders such as comp70, comp121, comp134, comp135, and comp136 are spatially similar, while those of weak binders such as comp13, comp15, and comp60 show more spatial variation.

### Hydrogen Bonds

Hydrogen bonds are critical to the binding interactions between the peptidomimetics and STAT3. Figure 6 shows all the residues that are involved in hydrogen bonds with at least one peptidomimetic. Residues Lys591, Arg609, Ser611, Glu612, and Ser613 are involved in the hydrogen bond interactions and form the phosphate-binding pocket (sub-pocket-1) where the phosphate group of pTyr residue (or its surrogate) in each peptidomimetic binds. Three sub-pockets in the binding site of the SH2 domain are also involved in hydrogen-bonding interactions. Residues Glu638, Tyr640, and Gln644 form sub-pocket-2, residues Gly656, Lys658, and Tyr657 form sub-pocket-3, and residues Trp623, Ile659, and Met660 flank sub-pocket-4.

The residues of the SH2 domain which participate in hydrogen bonds with a specific peptidomimetic and the hydrogen bond occupancy involving those residues and the peptidomimetic are shown in Figure 7. The occupancy plots in Figure 7 also show that the strong binders such as comp70, comp121, comp134, and comp136 form hydrogen bonds with more than 5 residues of the SH2 domain, and the weak binders such as comp13 and comp15 form hydrogen bonds with 3 and 4 residues respectively. Another weak-affinity peptidomimetic comp60 forms hydrogen bonds with 6 different residues but all of these residues surround the phosphate-binding pocket (sub-pocket-1). This means that, in the case of comp60, while the pTyr residue binds tightly to the sub-pocket-1, the rest of the peptidomimetic is not involved in stable hydrogen bond interactions. A couple of strong binders, comp135 and comp140, form hydrogen bonds with 4 residues each. Since the conformation of comp140 is unstable (as evident by the RMSF value) and we ignore hydrogen bonds if they are present in less than 50% of the conformations in the molecular dynamics trajectory, hydrogen bond interactions with fewer residues of the SH2 domain is expected. In the case of comp135, however, the RMSF value is low (1.22 Å). We postulate that comp135 may have an alternate and more stable bound conformation similar to the conformation of comp134.

### Binding Affinity

The binding affinity value reflects the thermodynamic stability of the binding interactions between a peptidomimetic and the SH2 domain of STAT3. In a computational modeling study such as this, a large positive correlation between the experimental binding affinities and estimated binding affinities is desired. A high correlation allows accurate prediction of strong and weak binders. We used binding energy function described by equation (2) to estimate the binding affinity values in four different schemes: A. entropic component (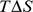) was ignored and the non-entropic component was computed using MMGBSA, B. entropic component (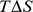) was ignored and the non-entropic component was computed using MMPBSA, C. entropic component (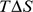) was included and the non-entropic component was computed using MMGBSA, and D. entropic component (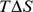) was included and the non-entropic component was computed using MMPBSA. The experimental binding affinities were calculated from the IC_50_ values using the function
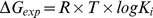
(3)where, K*_i_* = 
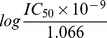
 mol, R (Gas constant) = 0.001986 kcal K^-1^ mol^-1^, T = 298 K, and IC_50_ values are in nM. The Cheng-Prusoff estimation [85] of K*_i_* from IC_50_ was done using a K*_d_* of 150 nM for FAM-Ala-pTyr-Leu-Pro-Gln-Thr-Val-NH_2_ [86] and a concentration of 10 nM in the fluorescence polarization assay used to evaluate the binding affinities of the 12 peptidomimetics [23].

Figure 8 (top) plots Pearson’s correlation coefficient (R), that measures the correlation between the experimental and estimated binding affinities, versus the length (2 ns, 4 ns, 6 ns, 8 ns, and 10 ns) of molecular dynamics simulation. Note that the binding affinities are computed averaged over the snapshots of the molecular dynamics simulation. From the figure, it is clear that, for all four affinity estimation schemes, the value of R increases with the increase in the length of molecular dynamics simulation. Out of the four schemes, the best correlation coefficient values were observed for the scheme D (cyan) which estimates affinity as a sum of the entropic component and the MMPBSA-based non-entropic component of the energy function. The maximum observed value of R is 0.63 which was computed using scheme D and 10 ns molecular dynamics simulation trajectories.

The estimated binding affinities, for the 12 peptidomimetics, obtained using scheme D are shown in Figure 8 (bottom). For each peptidomimetic, multiple values of the binding affinities that correspond to different lengths of molecular dynamics simulation are shown. It is clear that the affinity values converge as the length of simulation increases. The affinity values, derived from the 10 ns molecular dynamics trajectories, correspond to the R value of 0.63 as described above. Since the R value is large, not surprisingly, weak binders such as comp13, comp15, and comp60 have higher estimated affinity values, the value for comp15 (IC_50_> 100,000 nM) being the highest (−18.22 kcal/mol). Similarly, the binding affinity values for strong binders such as comp70, comp121, comp134, and comp136 are low, the value for comp70 (IC_50_ = 190 nM) being the lowest (−45.40 kcal/mol).

### Binding Modes

The conformations in Figure 5 show the presence of two binding modes that have also been described in previous computational modeling studies [24], [69]: the bent mode and the extended mode. All or some of the representative conformations for comp70, comp135, comp140, and comp142 display the bent mode where the phosphate group sits in sub-pocket 1 and the peptidomimetic bends such that the Gln (or its derivative) residue of the peptidomimetic sits in sub-pocket 2. In the extended mode, as seen in all or some of the representative conformations for comp134, comp136, comp140, and comp142, the phosphate group sits in sub-pocket 1 and the backbone extends such that Gln (or its derivative) residue of the peptidomimetic sits in sub-pocket 3. Apart from the bent and the extended modes, a novel binding mode was observed. The five representative conformations of comp121 display what we term a wedged mode. In this mode, while the phosphate group binds to the sub-pocket-1, the other end of the peptidomimetic is wedged in a groove formed by two loops of the SH2 domain described by residues 623–629 and residues 656–668.

The binding modes are shown in detail in Figures 9, 10, and 11. Both cartoon and surface representations of the SH2 domain are shown. The labeled yellow residues of the SH2 domain are involved in hydrogen bond interactions and the hydrogen bonds are shown with dashed black lines. The surface of the SH2 domain is colored using the Coulombic surface coloring scheme in the Chimera software package. The surface is characterized by electrostatic potentials ranging from positive electrostatic potential (blue surface) to a negative potential (red surface). The bent mode is displayed by the peptidomimetic comp70 (Figure 9), the extended mode is displayed by comp134 (Figure 10), and the wedged mode is displayed by comp121 (Figure 11). The binding affinities of the three peptidomimetics, experimental as well as computed, are high (low 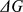 and IC_50_ values) and, as shown in Figure 4, the RMSF values for comp70 (0.98 Å), comp134 (0.95 Å), and comp121 (0.91 Å) are the lowest out of the RMSF values for the 12 peptidomimetics. Thus, these three compounds present a strong evidence that there are three possible modes in which peptidomimetics can tightly bind to the SH2 domain.

As expected, all three binding modes include multiple hydrogen bonds connecting the phosphate group to sub-pocket-1. The amino acids forming sub-pocket-1 create a strong positive electrostatic potential which thus tightly binds the negatively charged phosphate group in all peptidomimetics. In the bent mode (Figure 9), the Gln residue of comp70 binds to the sub-pocket-2 and forms multiple hydrogen bonds with residues Tyr640 and Gln644 of the SH2 domain that flank sub-pocket-2. The binding interactions are also stabilized by the hydrogen bonds formed between the carbonyl oxygen of the Haic group and residue Tyr657 of the SH2 domain. A similar interaction was observed between a carbonyl oxygen of pTyr-Asp-Lys-Pro-His and Tyr651 in the crystal structure of STAT1 [87]. In the extended mode (Figure 10), the carbonyl oxygen of the Leu at pTyr+1 position forms hydrogen bond with Tyr657 and the side chain amide group of the Gln-mimic residue at the C-terminus of the peptidomimetic forms hydrogen bonds with the main chain C = O of Gly656 and the backbone NH groups of Lys658 and Ile659. In the newly discovered wedged mode (Figure 11), the carbonyl oxygen of the Leu residue forms hydrogen bond with Trp623 which lies on the loop formed by residues 623–629 and the carbonyl oxygen of methanoproline is involved in a hydrogen bond with the side chain OH of Tyr657. The driving force for this binding mode appears to be hydrophobic contact between the C-terminal benzene ring and residues of loops 623–629 and 656–658 as well as a hydrogen bond between the benzylamide NH and the main chain C = O of Met660. Interestingly, the side chain amide group of Gln does not appear to interact directly with the protein.

## Discussion

Transcription factor STAT3 is an important target protein that is involved in a multitude of human cancers. In this work, we focused on a specific set of 12 peptidomimetic compounds that mimic the pTyr-Xaa-Yaa-Gln recognition motif and were designed to bind with the SH2 domain of STAT3 and prevent its dimerization which is a critical event leading up to the transcription of anti-apoptotic genes. Experimental binding affinities of the peptidomimetics were measured using fluorescence polarization and a range of affinity values were observed for the 12 peptidomimetics. Binding affinities for the peptidomimetics, expressed as IC_50_ values, range from 39 nM for a strong binder to over 100,000 nM for a weak binder. Since experimental structures of the complexes formed between the peptidomimetics and the SH2 domain are unavailable, we used a computational strategy to model the complexes.

Our modeling strategy proceeded in two steps. In the first, we generated docked conformations of the peptidomimetics using a computational AutoDock-based incremental docking protocol that was developed by us for docking large compounds in a fast and accurate manner [66]. The peptidomimetics in our dataset are all large compounds with the number of rotatable bonds ranging from 9 to 22. In the second step of our modeling strategy, we selected the best docked conformation and then ran molecular dynamics simulations of the complex in a solvated box. Molecular dynamics simulations served multiple purposes. The flexibility of the SH2 domain was taken into account, fluctuations of the bound conformations over the length of molecular dynamics simulation were computed, and finally, rigorous estimates of binding affinities, as a sum of normal-mode analysis based entropic component and MMPB/GBSA based non-entropic component, were computed. Accurate estimates of binding affinities are very important for differentiating strong binders from weak binders, and therefore, a positive correlation between the experimental binding affinities and estimated binding affinities is desired. Our two-step modeling strategy resulted in a high positive correlation (R = 0.63) between the experimental and estimated affinities.

For each of the 12 peptidomimetics, we performed molecular dynamics simulations for a production length of 10 ns. The trajectory data for each simulation was output at 10 ps. Thus, we obtained 1000 conformations for each peptidomimetic in complex with the SH2 domain. The average fluctuation of the conformations of each peptidomimetic was measured as RMSF (root mean square fluctuation) values. The weak binders displayed larger fluctuation as compared to the strong binders. A clustering of the conformations showed the preferred binding modes of the peptidomimetics. Three strong binders, with IC_50_ values equal to 190 nM (comp70), 83 nM (comp134), and 68 nM (comp121), displayed three different but stable binding modes: the bent mode, the extended mode, and the wedged mode respectively. The peptidomimetics in these three binding modes showed very small (<1.0 Å) conformational fluctuations in the molecular dynamics simulations, a large number of stable hydrogen bond interactions with the residues of the SH2 domain, and the estimated binding affinities value were low in accordance with the experimental binding affinities.

Previous modeling studies related to SH2 domain binding have proposed the bent and the extended binding modes [24], [69]. In this paper, we propose a new binding mode which we term the wedged mode. In the wedged mode, the peptidomimetic (comp121) binds to the SH2 domain such that the negatively charged phosphate group of the pTyr residue sits inside a pocket which has a positive electrostatic potential and the C-terminal benzyl group gets wedged in a cavity formed by two loops of the SH2 domain described by the residues 623–629 and 656–668 respectively. Apart from the stable hydrogen bond interactions with the residues in the phosphate-binding pocket, hydrogen bonds also exist between the peptidomimetic and residues on the two loops. The RMSF value for the 1000 conformations of the comp121 is 0.91 Å and is the lowest among the RMSF values for the 12 peptidomimetics.

Despite the overall success of modeling strategy as described in this paper, there were exceptions to the observed trends. For example, in the case of comp140 which is a relatively strong binder (IC_50_ = 105 nM), we obtained a large RMSF value and estimated binding affinities that are comparable to those of weak binders. This anomaly could be attributed to an inaccurate starting docked conformation of the peptidomimetic. In the molecular dynamics simulation, an inaccurate starting docked conformation would result in trajectory that leads to inaccurate estimation of binding affinity. It should be noted that computational docking of large ligands such as peptidomimetics in our dataset is extremely challenging. Although our incremental docking protocol improves docking of large ligands, more work needs to be done in this area.

The computational modeling strategy described in this paper and the subsequent data analysis, nonetheless, reveals important aspects of the peptidomimetic binding to the SH2 domain of STAT3. Not only were we able to estimate binding affinities that were well correlated with experimental binding affinities, we were also able to identify binding modes, including a novel wedge mode, that result in stable binding interactions. A typical peptidomimetic drug design process that is based on a specific motif involves designing peptidomimetics with diverse chemical modifications. Accurate estimation of binding affinities using our method could help in predicting which modifications could lead to strong binding. The knowledge gained by this study could also be used to improve the design of the peptidomimetics by better targeting the sub-binding-pockets identified in this paper with structural modifications or conformational restraints. The proposed novel wedge binding mode could prove very useful in this regard.

## Supporting Information

Section S1 **Incremental Docking Details.**
(PDF)

Section S2 **Modeling approach validation study.**
(PDF)

Figure S1 **MD simulation of comp1-SH2 complex.** Total energy (ETOT), potential energy (EPTOT), temperature (TEMP), and pressure (PRES) over the course of 10 ns MD trajectory.
(PNG)

Figure S2 **MD simulation of comp13-SH2 complex.** Total energy (ETOT), potential energy (EPTOT), temperature (TEMP), and pressure (PRES) over the course of 10 ns MD trajectory.
(PNG)

Figure S3 **MD simulation of comp15-SH2 complex.** Total energy (ETOT), potential energy (EPTOT), temperature (TEMP), and pressure (PRES) over the course of 10 ns MD trajectory.
(PDF)

Figure S4 **MD simulation of comp60-SH2 complex.** Total energy (ETOT), potential energy (EPTOT), temperature (TEMP), and pressure (PRES) over the course of 10 ns MD trajectory.
(PNG)

Figure S5 **MD simulation of comp70-SH2 complex.** Total energy (ETOT), potential energy (EPTOT), temperature (TEMP), and pressure (PRES) over the course of 10 ns MD trajectory.
(PNG)

Figure S6 **MD simulation of comp108-SH2 complex.** Total energy (ETOT), potential energy (EPTOT), temperature (TEMP), and pressure (PRES) over the course of 10 ns MD trajectory.
(PNG)

Figure S7 **MD simulation of comp121-SH2 complex.** Total energy (ETOT), potential energy (EPTOT), temperature (TEMP), and pressure (PRES) over the course of 10 ns MD trajectory.
(PNG)

Figure S8 **MD simulation of comp134-SH2 complex.** Total energy (ETOT), potential energy (EPTOT), temperature (TEMP), and pressure (PRES) over the course of 10 ns MD trajectory.
(PNG)

Figure S9 **MD simulation of comp135-SH2 complex.** Total energy (ETOT), potential energy (EPTOT), temperature (TEMP), and pressure (PRES) over the course of 10 ns MD trajectory.
(PNG)

Figure S10 **MD simulation of comp136-SH2 complex.** Total energy (ETOT), potential energy (EPTOT), temperature (TEMP), and pressure (PRES) over the course of 10 ns MD trajectory.
(PNG)

Figure S11 **MD simulation of comp140-SH2 complex.** Total energy (ETOT), potential energy (EPTOT), temperature (TEMP), and pressure (PRES) over the course of 10 ns MD trajectory.
(PNG)

Figure S12 **MD simulation of comp142-SH2 complex.** Total energy (ETOT), potential energy (EPTOT), temperature (TEMP), and pressure (PRES) over the course of 10 ns MD trajectory.
(PNG)

Figure S13 **Protein-ligand complex with PDB ID 1BM2.** The experimental conformation (green) and the modeled conformation (yellow) of the ligand are shown in stick representation and the protein is shown in surface representation.
(PNG)

Figure S14 **Protein-ligand complex with PDB ID 1CJ1.** The experimental conformation (green) and the modeled conformation (yellow) of the ligand are shown in stick representation and the protein is shown in surface representation.
(PNG)

Figure S15 **Protein-ligand complex with PDB ID 1IJR.** The experimental conformation (green) and the modeled conformation (yellow) of the ligand are shown in stick representation and the protein is shown in surface representation.
(PNG)

Figure S16 **Protein-ligand complex with PDB ID 1SKJ.** The experimental conformation (green) and the modeled conformation (yellow) of the ligand are shown in stick representation and the protein is shown in surface representation.
(PNG)

Figure S17 **Protein-ligand complex with PDB ID 1BKM.** The experimental conformation (green) and the modeled conformation (yellow) of the ligand are shown in stick representation and the protein is shown in surface representation.
(PNG)

Figure S18 **Protein-ligand complex with PDB ID 1IS0.** The experimental conformation (green) and the modeled conformation (yellow) of the ligand are shown in stick representation and the protein is shown in surface representation.
(PNG)

Figure S19 **Protein-ligand complex with PDB ID 1A08.** The experimental conformation (green) and the modeled conformation (yellow) of the ligand are shown in stick representation and the protein is shown in surface representation.
(PNG)

Figure S20 **Protein-ligand complex with PDB ID 1SHD.** The experimental conformation (green) and the modeled conformation (yellow) of the ligand are shown in stick representation and the protein is shown in surface representation.
(PNG)

Figure S21 **Protein-ligand complex with PDB ID 1SPS.** The experimental conformation (green) and the modeled conformation (yellow) of the ligand are shown in stick representation and the protein is shown in surface representation.
(PNG)

Figure S22 **Protein-ligand complex with PDB ID 1ZFP.** The experimental conformation (green) and the modeled conformation (yellow) of the ligand are shown in stick representation and the protein is shown in surface representation.
(PNG)

Table S1 Number of atoms in MD simulation systems. MD simulations were performed on 12 systems each comprising of one of the 12 peptidomimetics in complex with the SH2 domain of STAT3 in a explicit solvent box. This table lists the number of atoms (Natoms) in each system.
(PNG)

Table S2 Validation accuracy. 10 protein-ligand complexes were identified for validation of our modeling approach to predict binding modes of peptidomimetics in complex with the SH2 domain of STAT3. This table lists the PDB IDs that correspond to the deposited experimental structures of the 10 complexes. The RMSD values between the modeled conformation and experimenal conformation of the ligands evaluate the accuracy of our modeling approach. N represents the number of rotatable bonds in the ligands.
(PNG)
