# Supplementary material for: Binding Modes of Peptidomimetics Designed to Inhibit STAT3
Source: PLoS One. 2012 Dec 12;7(12):e51603. doi: 10.1371/journal.pone.0051603 (PMC3520966; doi:10.1371/journal.pone.0051603)
Supplement: Section S1 — Incremental Docking Details. (PDF) [file pone.0051603.s001.pdf]

Supporting Information: Binding modes of peptidomimetics designed to inhibit STAT3

Ankur Dhanik[1], John S McMurray[2], Lydia E Kavraki[1*]
1 Department of Computer Science, Rice University, Houston, Texas, USA
2 Department of Experimental Therapeutics, University of Texas MD Anderson Cancer Center, Houston, Texas, USA
∗ **E-mail: kavraki@rice.edu**

S1 Incremental docking details

Our AutoDock-based incremental docking protocol [66], at each incremental step, explores a few rotatable bonds. A small fragment of a large ligand, with a small number of rotatable bonds, is first selected and its rotatable bonds are set active. Only the active rotatable bonds are explored in each docking operation. The fragment is docked to the protein using AutoDock [44,65] and few best-scoring conformations are selected. The selected conformations are grown by adding a few more rotatable bonds and the atoms that are directly rotated by them. A few of the rotatable bonds in the the grown fragments are set active and the grown fragments are subsequently docked using AutoDock. The above dock-select-grow-dock process is repeated until all the rotatable bonds in the ligand are explored. AutoDock is used in each step to explore only a few rotatable bonds and this makes the docking operation fast and accurate.

Each peptidomimetic in our dataset was docked to the SH2 domain of STAT3 using our incremental docking protocol. The first fragment was selected such that it contains the phosphotyrosine (pTyr) residue of the peptidomimetic. The phosphate group of the pTyr residue is known to bind to the sub-pocket formed by residues Lys591, Arg609, Ser611, Glu612, and Ser613. Our docking protocol makes sure that, at each incremental step, the fragment contains the phosphate group and the phosphate group in the docked conformation of the fragment is spatially close to the sub-pocket. The atoms and bonds comprising the first fragment were selected in the following manner. A torsion tree was constructed rooted at the phosphorus atom (from the pTyr residue) such that an edge of the tree represents a rotatable bond and a node represents a set of atoms. Suppose there is an edge E from node A to B. Then B contains all atoms that are directly rotated by the rotatable bond corresponding to E. Suppose there are in total N edges in the torsion tree corresponding to N rotatable bonds in the peptidomimetic. The edges of the torsion tree were ranked from 1 to N based on the visit order of each edge upon a breadth-first traversal (starting at the root node) of the tree. The first fragment, thus, contained atoms and bonds that are directly rotated by the rotatable bonds ranked from 1 to $min(N, 6)$.

After each docking operation using AutoDock, 5 best-scoring docked conformations were selected. A docked conformation was considered better if it has low binding affinity (as estimated by AutoDock) and the phosphate group of the pTyr residue lies in its known binding sub-pocket. This was achieved by selecting 5 conformations with the lowest value of S, where

$$S = 0.25(P_d) + S_{AD}, \tag{1}$$

P_d is the squared distance of the phosphorus atom (in pTyr) from coordinates $(-8.42, 4.50, -6.09)$ that represent approximate center of the sub-pocket, and S_{AD} is the binding affinity estimated by AutoDock's energy function. The weight for P_d accounts for the 2.5kcal/mol of standard error in AutoDock's scoring function and $P_d > 10\text{Å}^2$ as a condition for the phosphate group lying outside the sub-pocket. The selected conformations were grown by adding atoms and bonds that are directly rotated by rotatable bonds ranked from $min(N, 6) + 1$ to $min(N, (min(N, 6) + 3))$. The rotatable bonds ranked from $min(N, 6) - 2$ to $min(N, (min(N, 6) + 3))$ were set active and the grown fragments were docked again. Thus, at each increment, a maximum of 6 rotatable bonds were explored.

For docking operations using AutoDock, the SH2 domain of STAT3 and each peptidomimetic in the dataset were processed in the following manner. The atoms of the peptidomimetic were assigned Gasteiger

charges. All non-polar hydrogen atoms and lone-pair charges were removed. The charge of each removed non-polar hydrogen was added to the carbon atom to which it is bonded and the charge of each lone pair was added to the atom associated with it. The protein atoms were also assigned Gasteiger charges. The non-polar hydrogen atoms and lone-pairs of the SH2 domain were processed in the same way as those of the ligand. Any bond in the peptidomimetic that is an amide bond, or is in a cycle, or rotates only hydrogen atoms was labeled non-rotatable, and the rest of the bonds were labeled rotatable. The AutoDock grid was specified such that it encompasses the full binding pocket of the SH2 domain that is involved in the dimerization of STAT3. The grid was centered at coordinates $(-5.22, -1.37, -0.43)$ and each dimension of the grid was set equal to 60Å. AutoDock parameters *ga_run* and *ga_num_evals* were set to 20 and $250,000$ during each docking operation. All other AutoDock parameters were set to their default values.
