# Supplementary material for: Binding Modes of Peptidomimetics Designed to Inhibit STAT3
Source: PLoS One. 2012 Dec 12;7(12):e51603. doi: 10.1371/journal.pone.0051603 (PMC3520966; doi:10.1371/journal.pone.0051603)
Supplement: Section S2 — Modeling approach validation study. (PDF) [file pone.0051603.s002.pdf]

Supporting Information: Binding modes of peptidomimetics designed to inhibit STAT3

Ankur Dhanik[1], John S McMurray[2], Lydia E Kavraki[1*]
1 Department of Computer Science, Rice University, Houston, Texas, USA
2 Department of Experimental Therapeutics, University of Texas MD Anderson Cancer Center, Houston, Texas, USA
∗ E-mail: kavraki@rice.edu

S2 Modeling approach validation study

In our recent work [66] we evaluated the accuracy of our incremental docking protocol by comparing modeled structures with experimentally-determined structures of protein-ligand complexes. Evaluating the accuracy of modeled structures of peptidomimetics in complex with the SH2 domain of STAT3 is, however, challenging because experimentally-determined structures of such complexes are unavailable in the Protein Data Bank (PDB). Therefore, to evaluate the accuracy of our modeling approach, we performed this validation study that compared modeled and experimental structures of complexes that are similar to the peptidomimetic-SH2 domain complexes. The accuracy of our modeling approach was measured as the RMSD distance between the modeled conformation of the ligand and the experimentally-determined conformation.

To create the validation dataset, first we extracted structures from the PDB database using a "SH2 domain" search query. The identified structures were then cross-referenced with the PDBbind database [77], a database of structures of protein-ligand complexes derived from the PDB. This resulted in structures of protein-ligand complexes that contain the SH2 domain. Since we are interested in modeling phosphotyrosine (pTyr)-based peptidomimetics, we extracted all the structures in which the ligand contains a phosphate group. It is to be noted that none of the structures contained a receptor from the STAT family. In our peptidomimetic dataset, the largest peptidomimetic has 22 rotatable bonds. We, therefore, filtered out all structures in which the ligand has more than 25 rotatable bonds. Out of the remaining 11 structures we eliminated structure with PDB ID 1FYR because it contains a very flexible ligand binding site that displayed large deformations in molecular dynamics (MD) simulation (a critical component of our modeling approach) and, thus, made the evaluation of RMSD arbitrary. The dataset for validation, therefore, contains structures of 10 protein-ligand complexes in which a peptide or peptidomimetic (containing a phosphate group) is bound to a SH2 domain.

Table S2 lists the 10 protein-ligand complexes in the validation dataset and the RMSD values computed between the modeled conformation and the experimental conformation deposited in the PDB. Figures S13-S22 show the conformations (modeled and experimental) of the ligands in complex with the protein as well as superimposed over each other. The modeled and experimental conformations of the ligand with IDs 1BM2, 1IJR, 1SKJ, 1BKM, 1IS0, 1A08, and 1SHD are close to each other. Even though modeled conformations of the ligands with IDs 1IS0 and 1SHD have high RMSD values, the modeled and experimental conformations as shown in Figures S18 and S20 are similar and target similar binding pockets. The modeled conformation of the ligand with ID 1ZFP has high RMSD value, but Figure S22 shows that fragments of the ligand in both modeled and experimental conformations target same binding pockets and the major spatial variation between conformations is only in the solvent exposed fragment of the ligand. The modeled conformation of the ligand with ID 1CJ1 also has high RMSD value. Although many atoms in the modeled and experimental conformations are close to each other, the high RMSD value is because of two terminal groups that have switched places in the modeled conformation (Figure S14). Finally the very high RMSD value of the modeled conformation of the ligand with PDB ID 1SPS shows that in spite of overall very accurate modeling of binding modes, there is scope for improvement. It is also extremely important to note that multiple binding modes of a ligand could exist in

nature; nonetheless our modeling approach is able to accurately predict the modes that have so far been experimentally observed.
