# Supplementary figures and images for: Binding Modes of Peptidomimetics Designed to Inhibit STAT3
Source: PLoS One. 2012 Dec 12;7(12):e51603. doi: 10.1371/journal.pone.0051603 (PMC3520966; doi:10.1371/journal.pone.0051603)

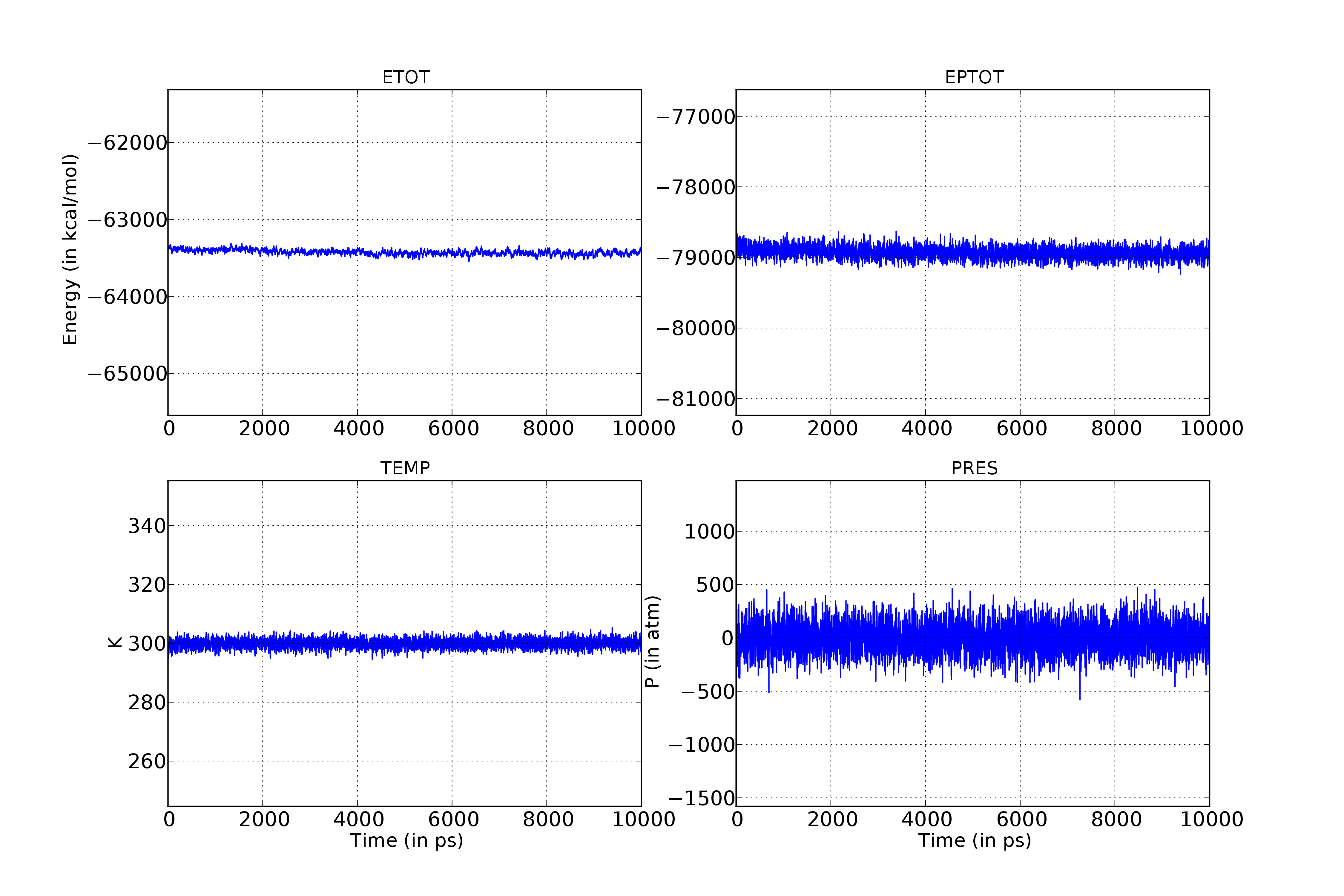

Supplement: Figure S1 — MD simulation of comp1-SH2 complex. Total energy (ETOT), potential energy (EPTOT), temperature (TEMP), and pressure (PRES) over the course of 10 ns MD trajectory. (PNG) [file pone.0051603.s003.png]

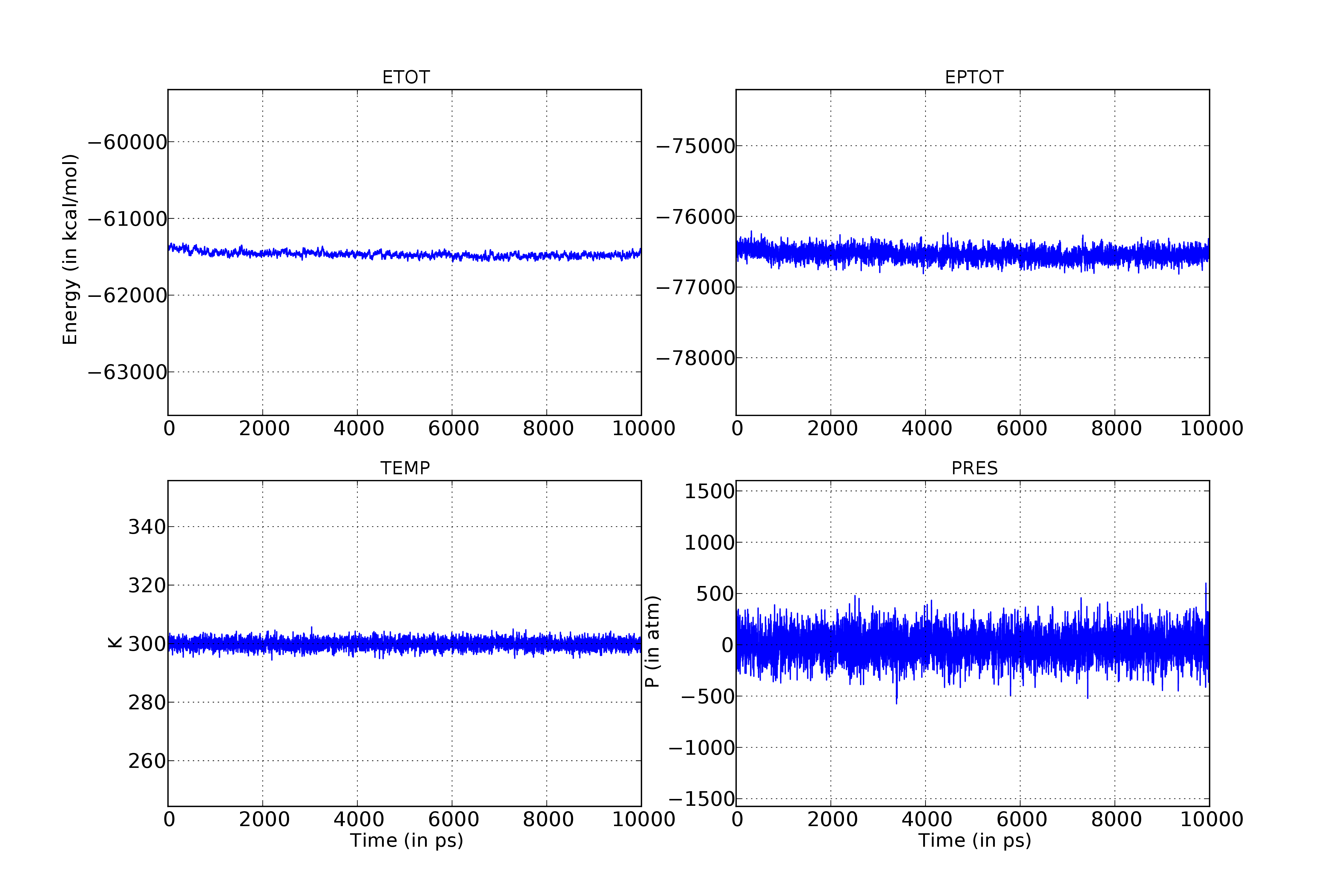

Supplement: Figure S2 — MD simulation of comp13-SH2 complex. Total energy (ETOT), potential energy (EPTOT), temperature (TEMP), and pressure (PRES) over the course of 10 ns MD trajectory. (PNG) [file pone.0051603.s004.png]

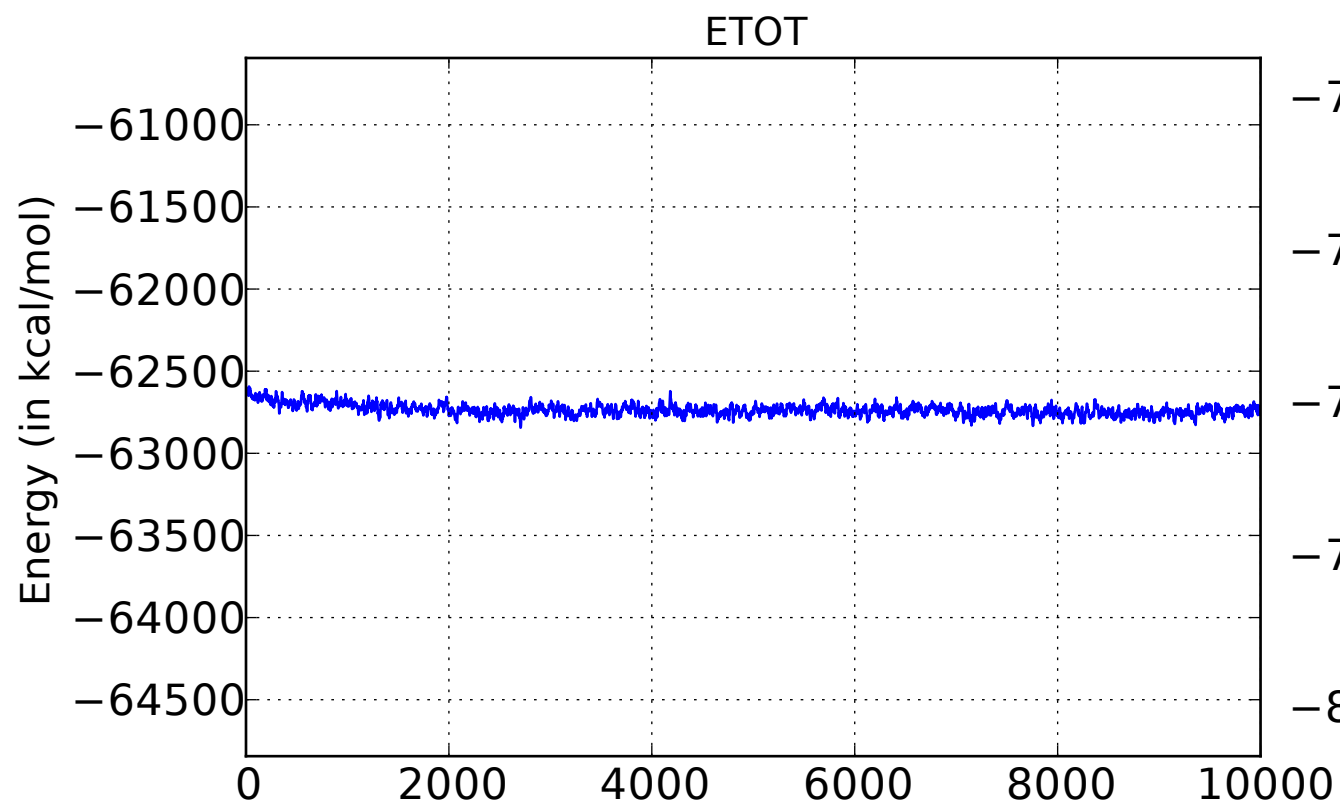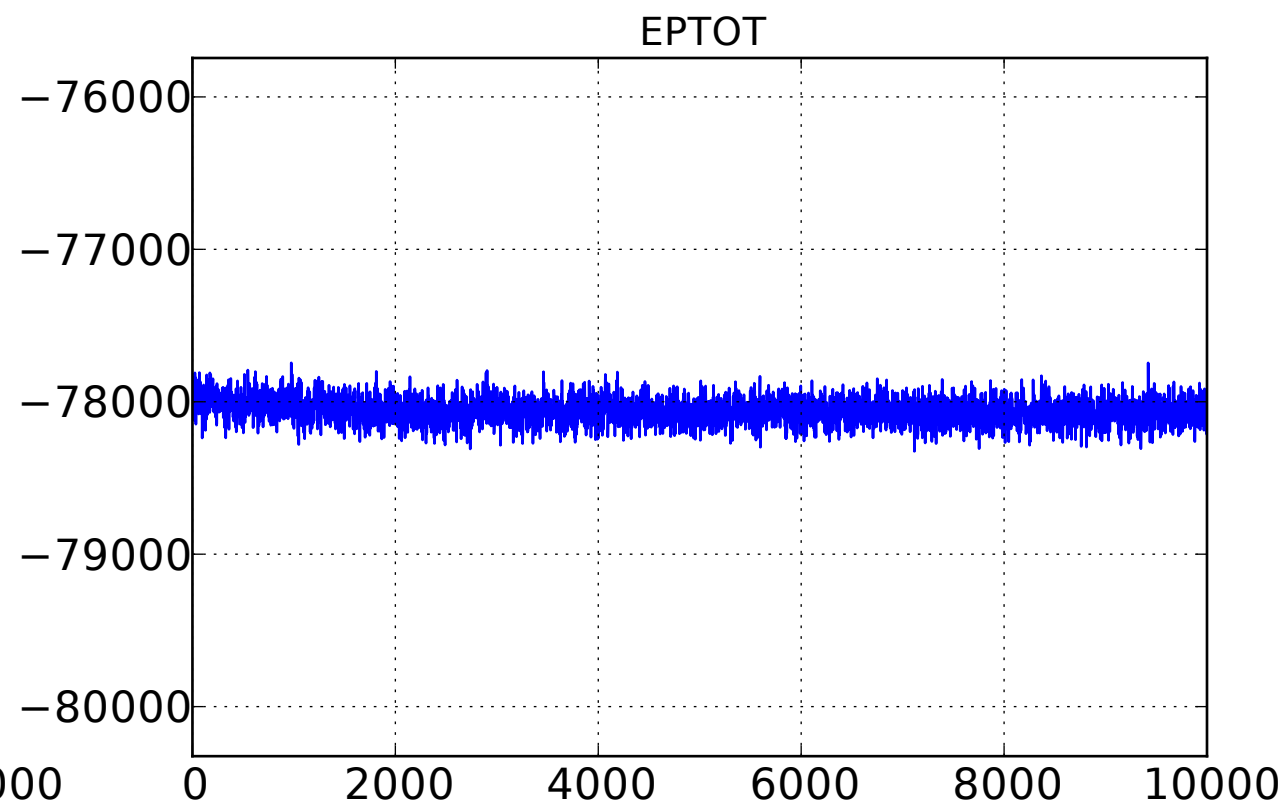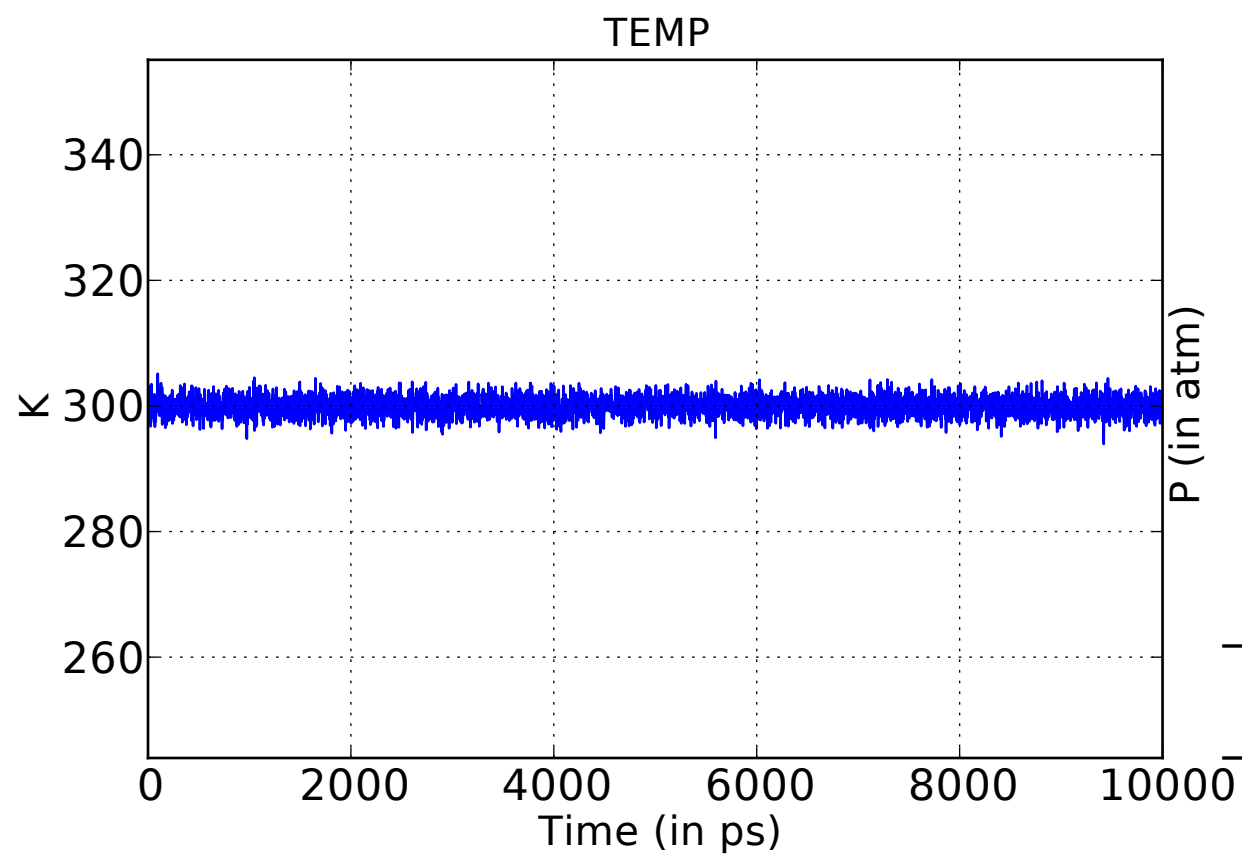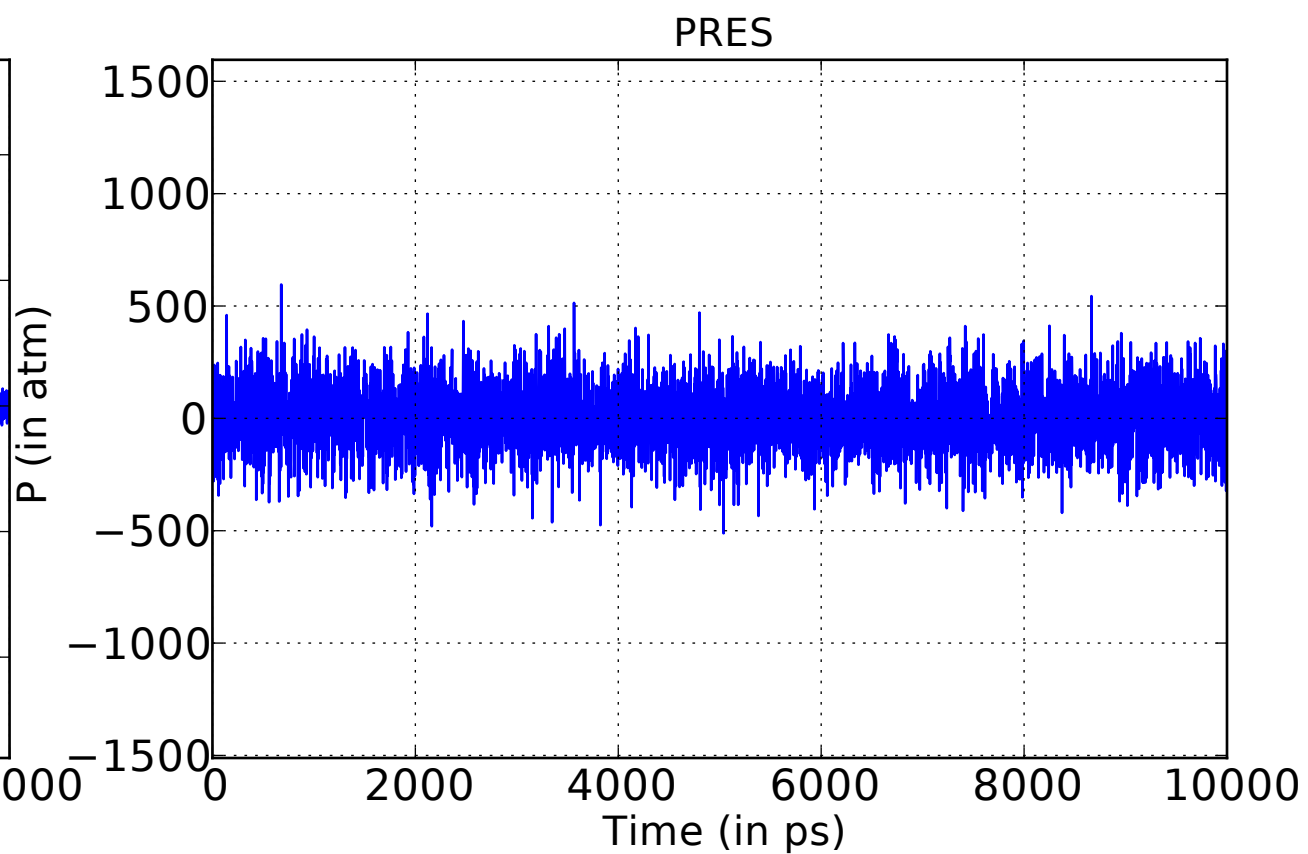

Supplement: Figure S3 — MD simulation of comp15-SH2 complex. Total energy (ETOT), potential energy (EPTOT), temperature (TEMP), and pressure (PRES) over the course of 10 ns MD trajectory. (PDF) [file pone.0051603.s005.pdf]

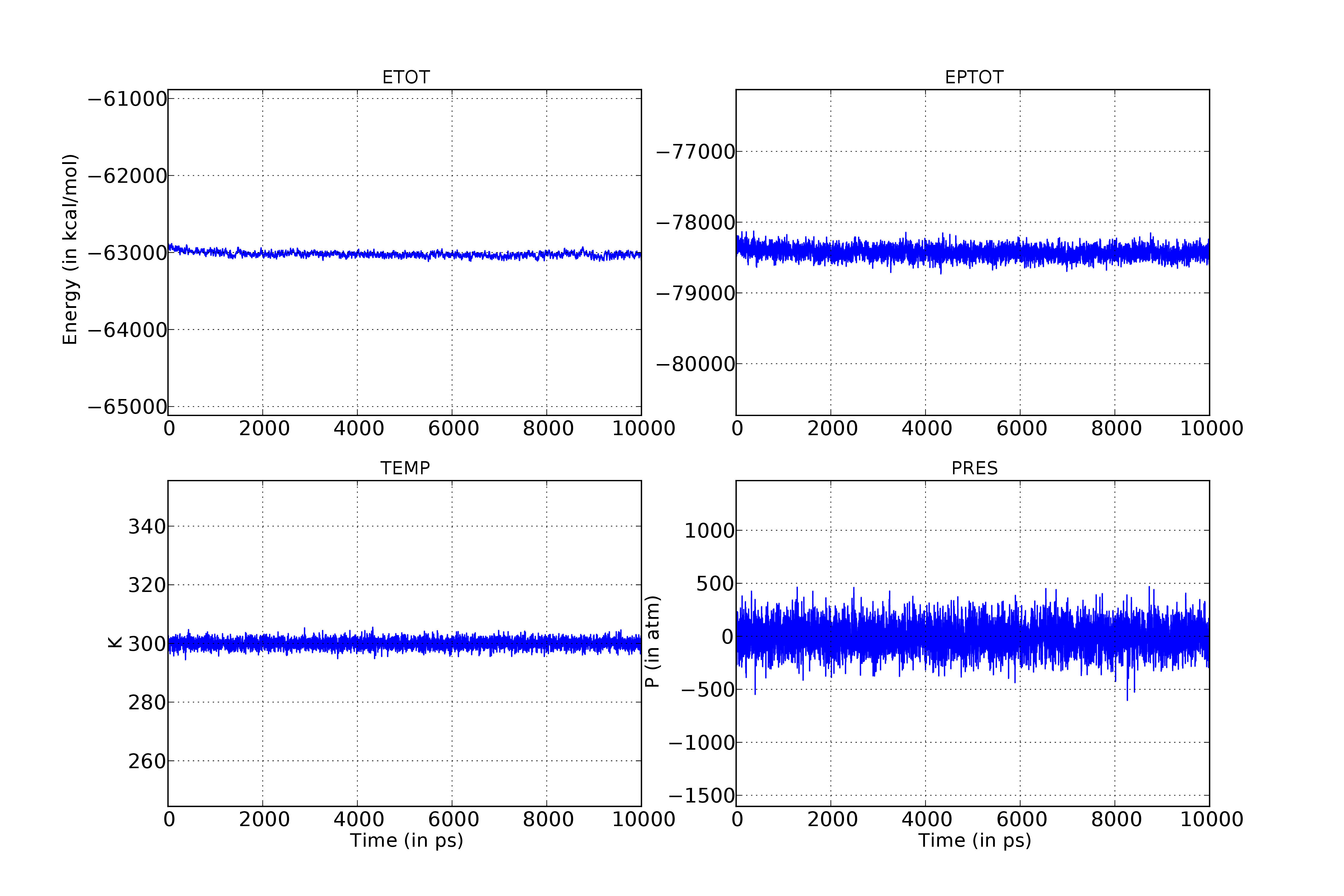

Supplement: Figure S4 — MD simulation of comp60-SH2 complex. Total energy (ETOT), potential energy (EPTOT), temperature (TEMP), and pressure (PRES) over the course of 10 ns MD trajectory. (PNG) [file pone.0051603.s006.png]

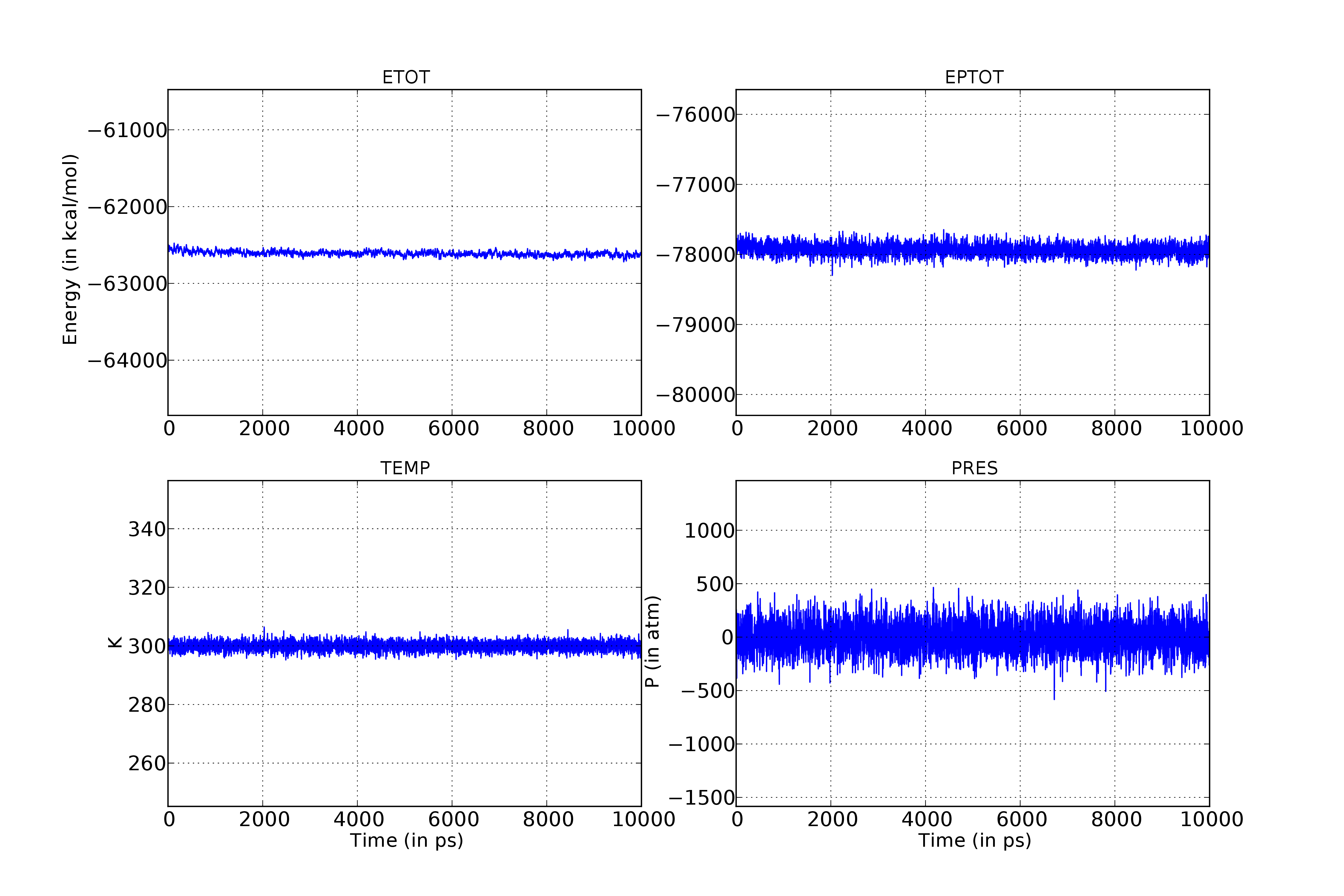

Supplement: Figure S5 — MD simulation of comp70-SH2 complex. Total energy (ETOT), potential energy (EPTOT), temperature (TEMP), and pressure (PRES) over the course of 10 ns MD trajectory. (PNG) [file pone.0051603.s007.png]

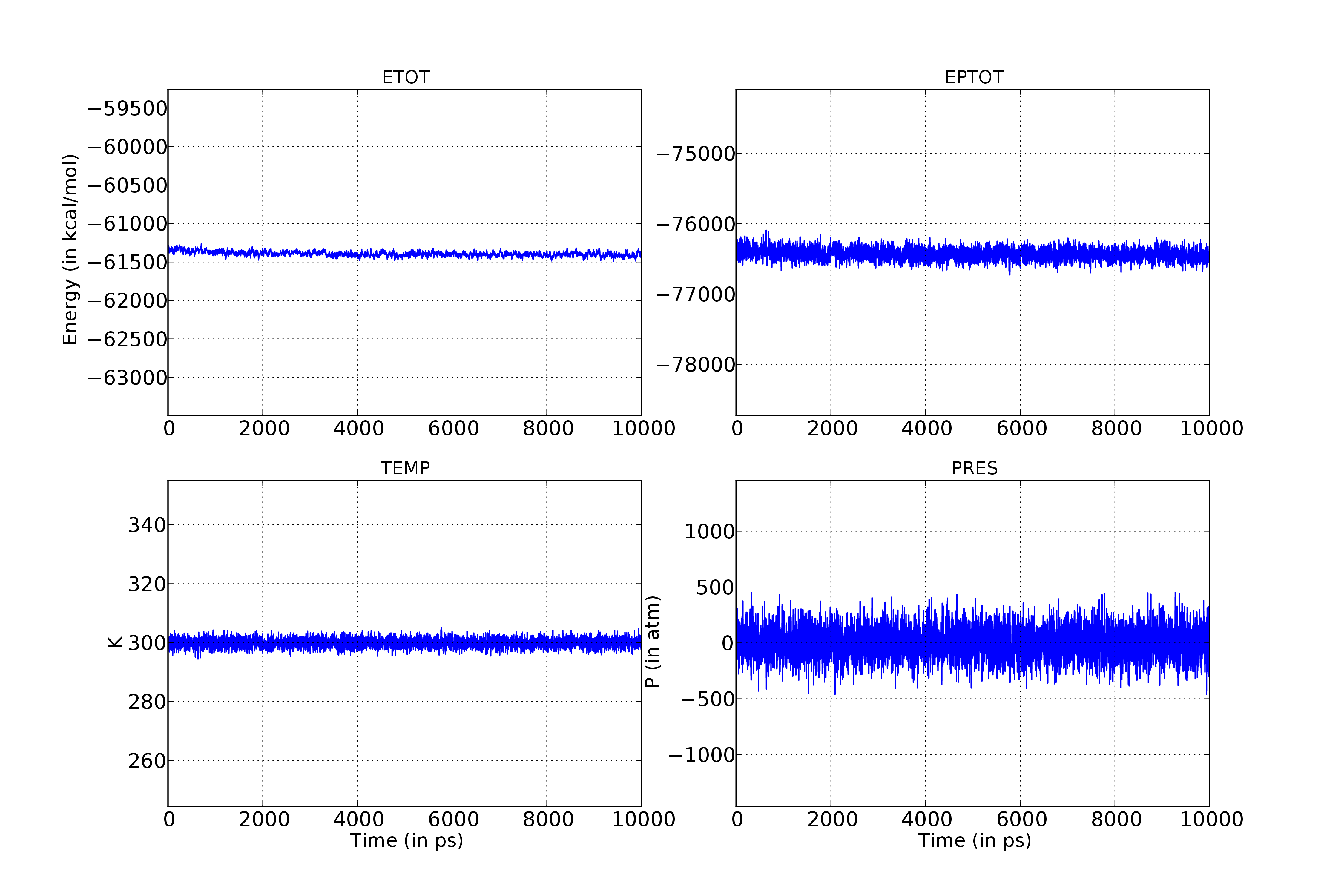

Supplement: Figure S6 — MD simulation of comp108-SH2 complex. Total energy (ETOT), potential energy (EPTOT), temperature (TEMP), and pressure (PRES) over the course of 10 ns MD trajectory. (PNG) [file pone.0051603.s008.png]

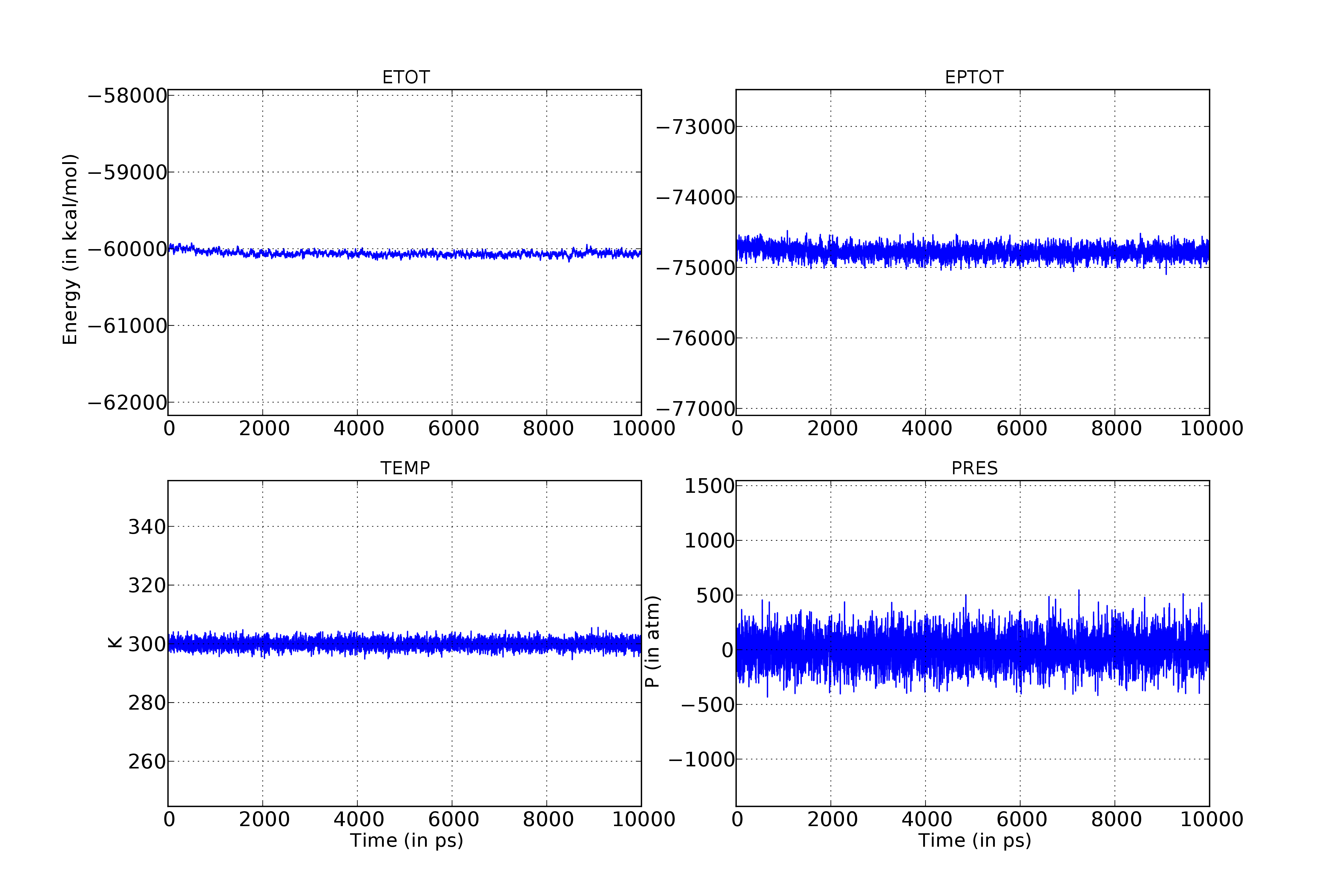

Supplement: Figure S7 — MD simulation of comp121-SH2 complex. Total energy (ETOT), potential energy (EPTOT), temperature (TEMP), and pressure (PRES) over the course of 10 ns MD trajectory. (PNG) [file pone.0051603.s009.png]

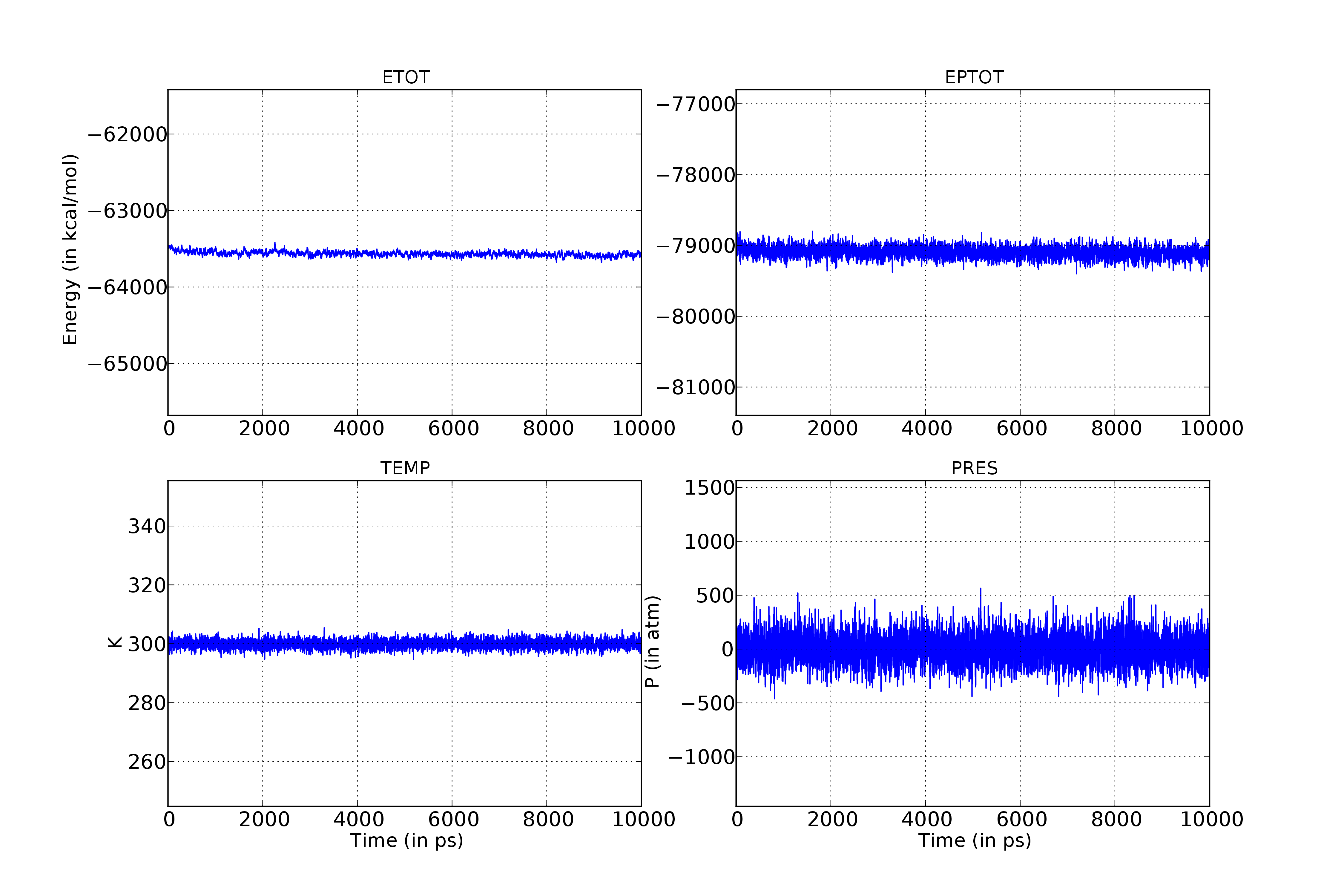

Supplement: Figure S8 — MD simulation of comp134-SH2 complex. Total energy (ETOT), potential energy (EPTOT), temperature (TEMP), and pressure (PRES) over the course of 10 ns MD trajectory. (PNG) [file pone.0051603.s010.png]

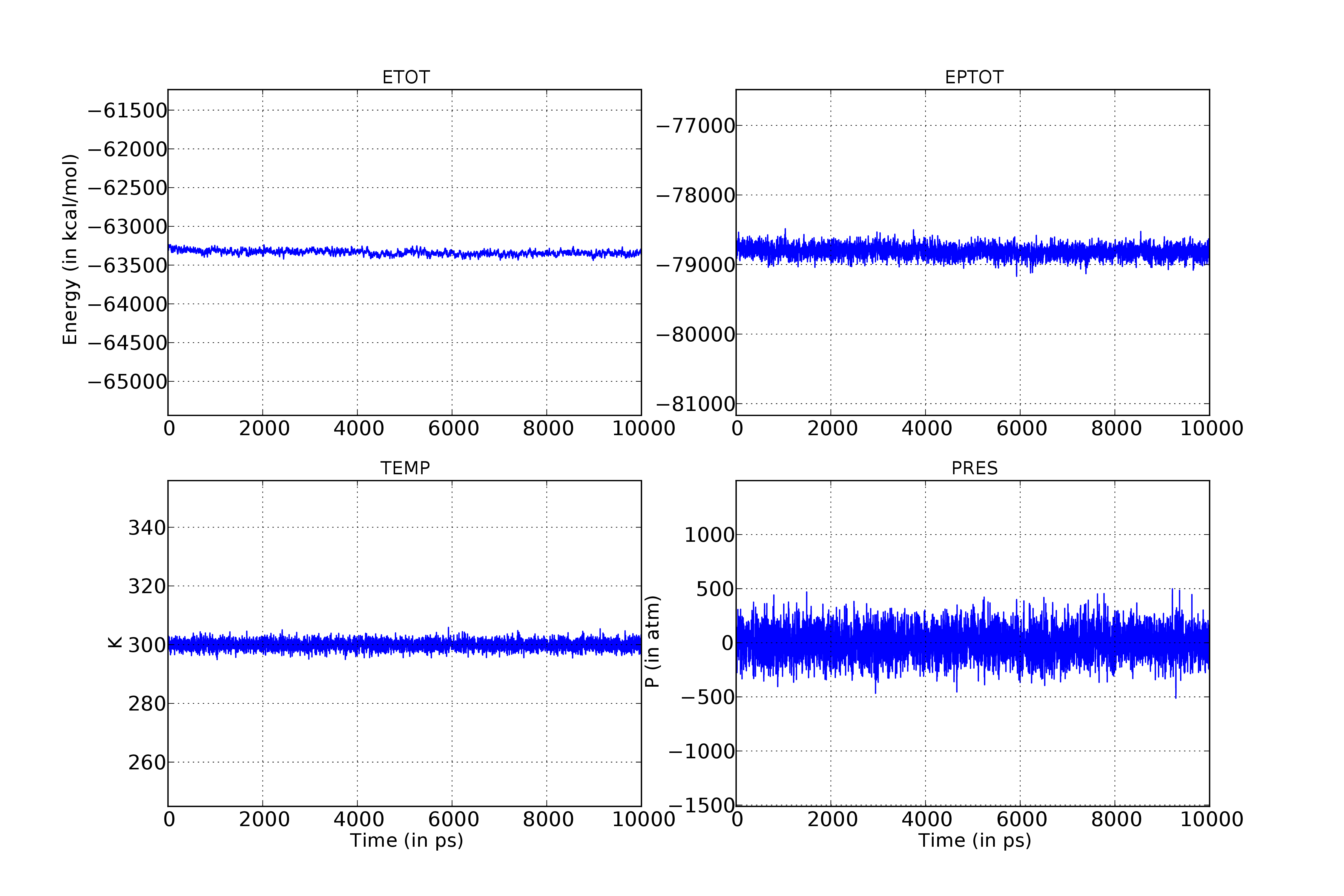

Supplement: Figure S9 — MD simulation of comp135-SH2 complex. Total energy (ETOT), potential energy (EPTOT), temperature (TEMP), and pressure (PRES) over the course of 10 ns MD trajectory. (PNG) [file pone.0051603.s011.png]

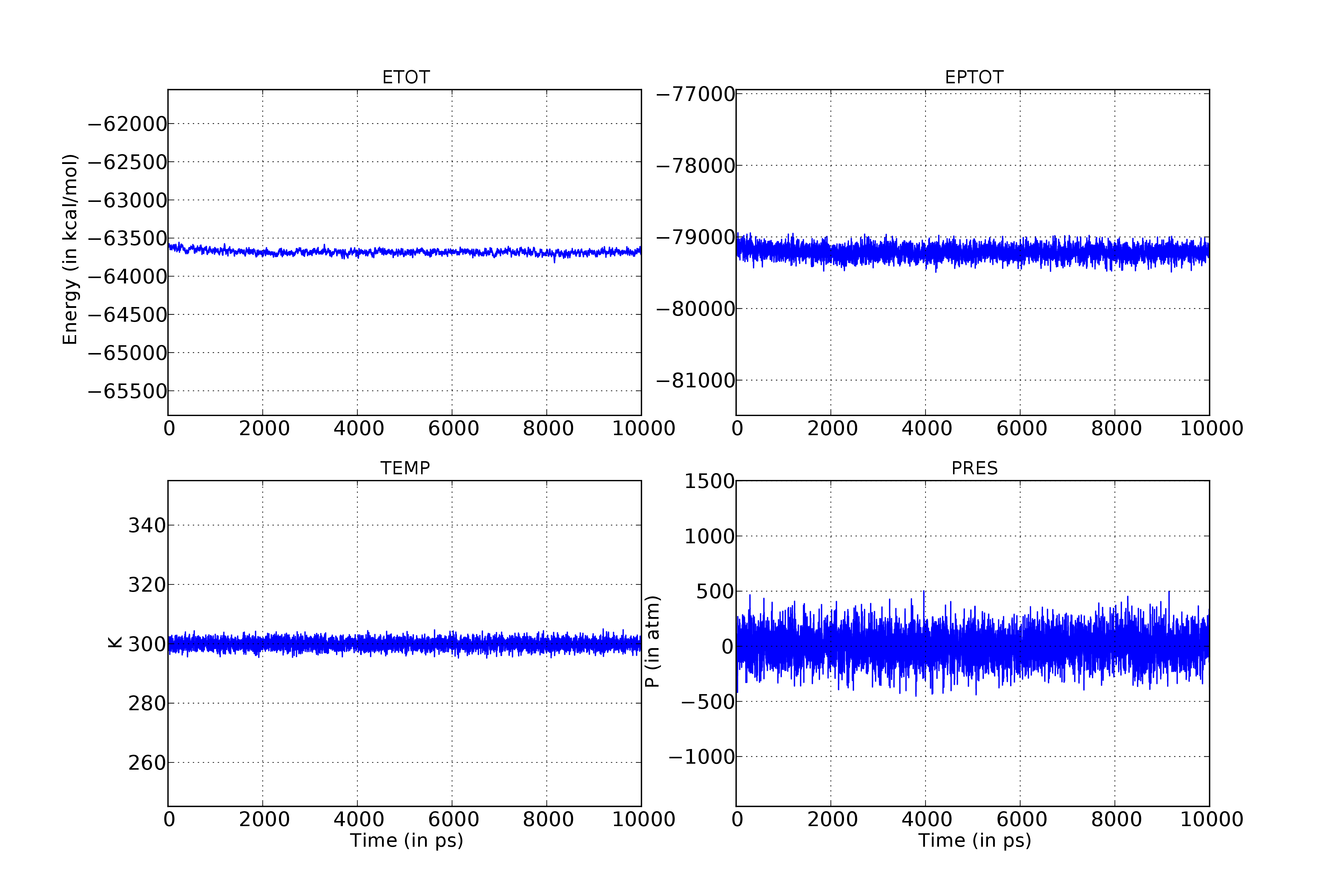

Supplement: Figure S10 — MD simulation of comp136-SH2 complex. Total energy (ETOT), potential energy (EPTOT), temperature (TEMP), and pressure (PRES) over the course of 10 ns MD trajectory. (PNG) [file pone.0051603.s012.png]

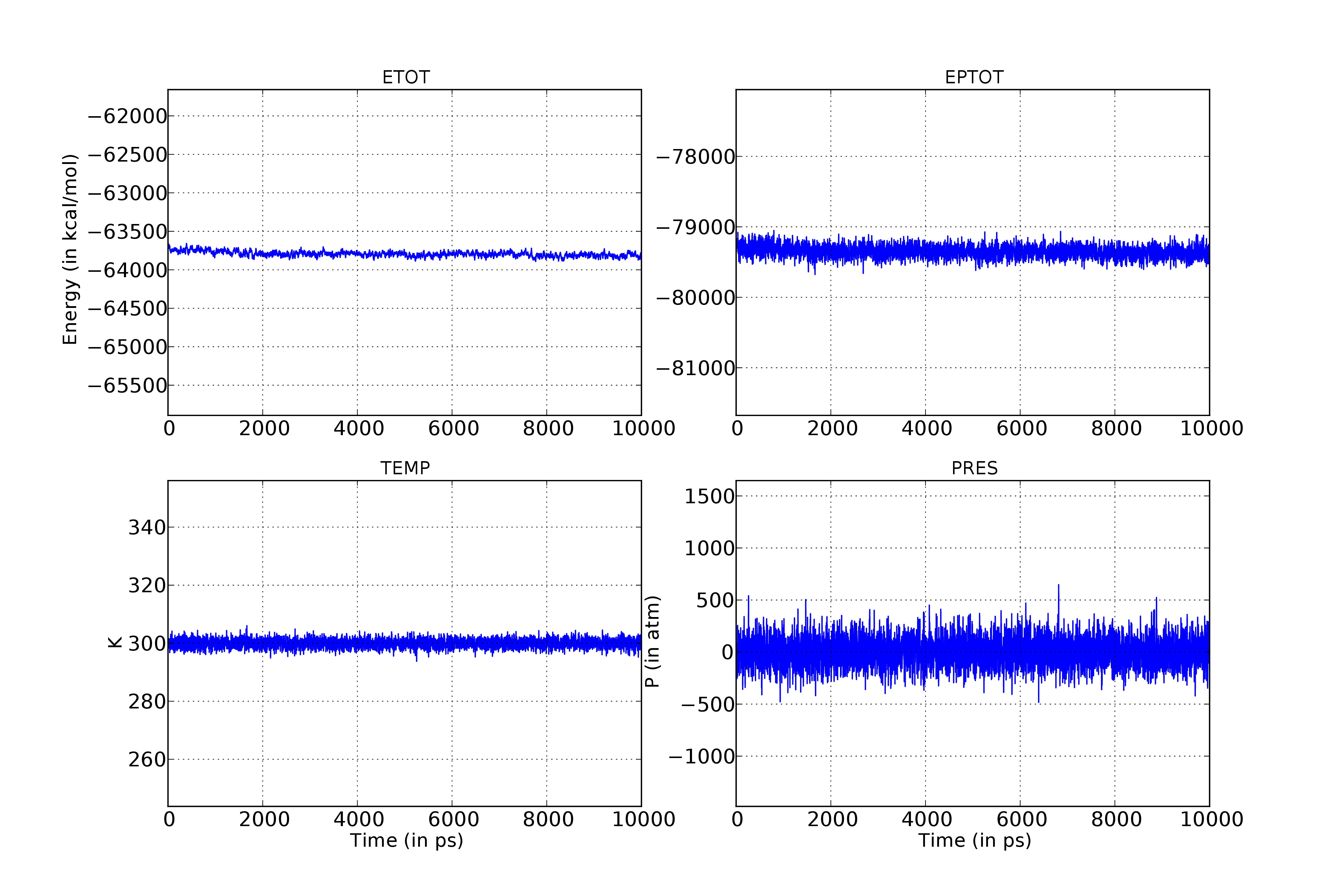

Supplement: Figure S11 — MD simulation of comp140-SH2 complex. Total energy (ETOT), potential energy (EPTOT), temperature (TEMP), and pressure (PRES) over the course of 10 ns MD trajectory. (PNG) [file pone.0051603.s013.png]

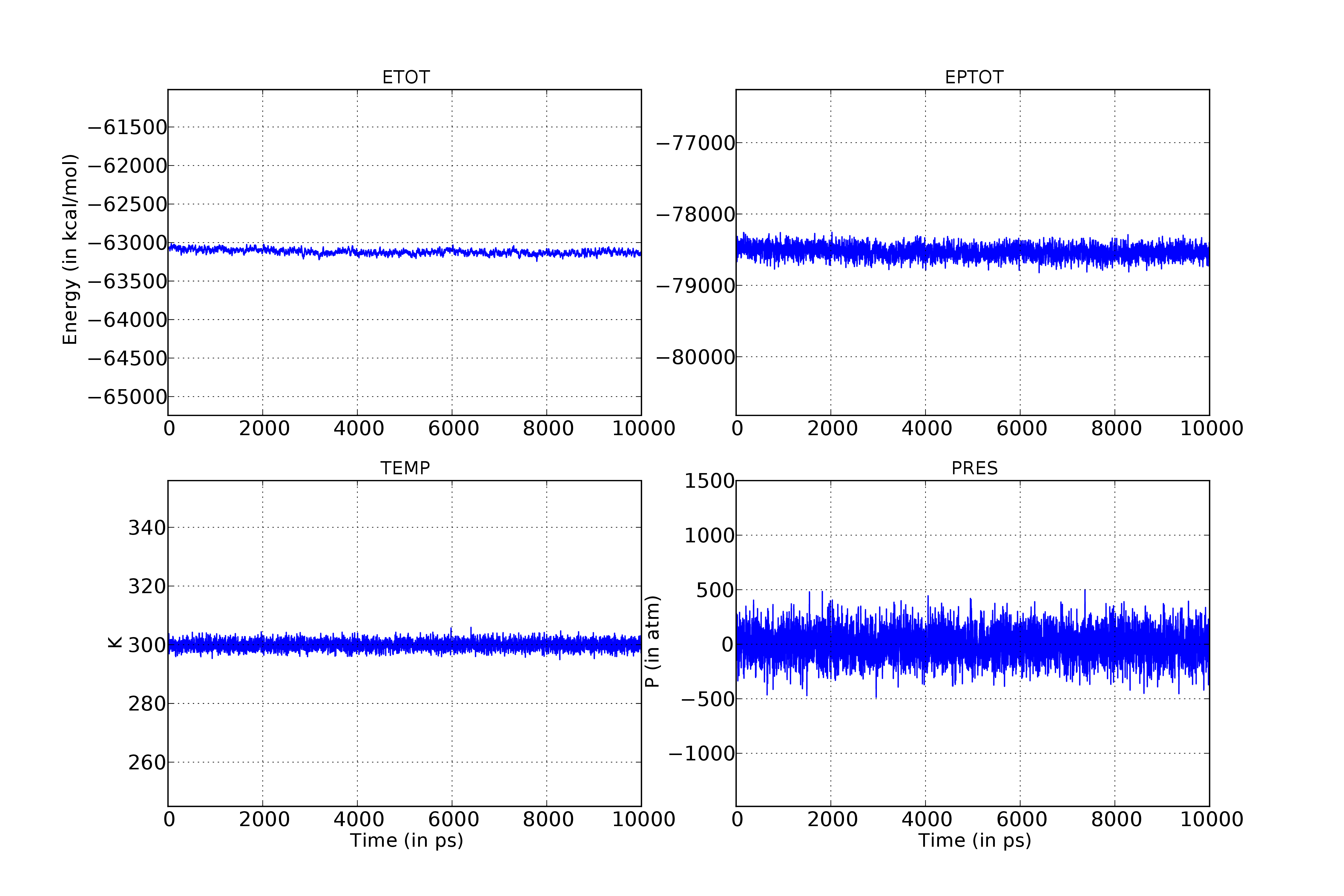

Supplement: Figure S12 — MD simulation of comp142-SH2 complex. Total energy (ETOT), potential energy (EPTOT), temperature (TEMP), and pressure (PRES) over the course of 10 ns MD trajectory. (PNG) [file pone.0051603.s014.png]

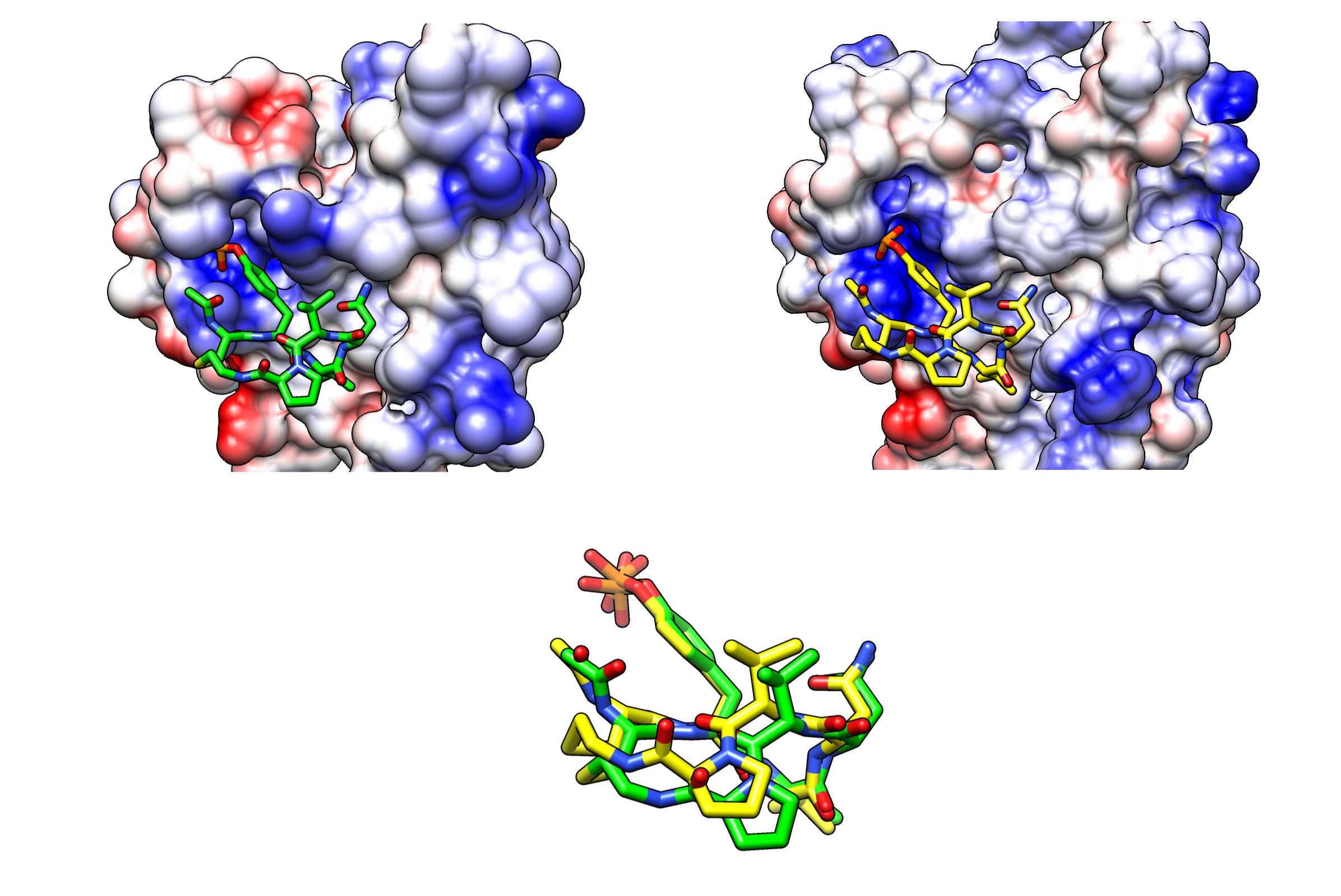

Supplement: Figure S13 — Protein-ligand complex with PDB ID 1BM2. The experimental conformation (green) and the modeled conformation (yellow) of the ligand are shown in stick representation and the protein is shown in surface representation. (PNG) [file pone.0051603.s015.png]

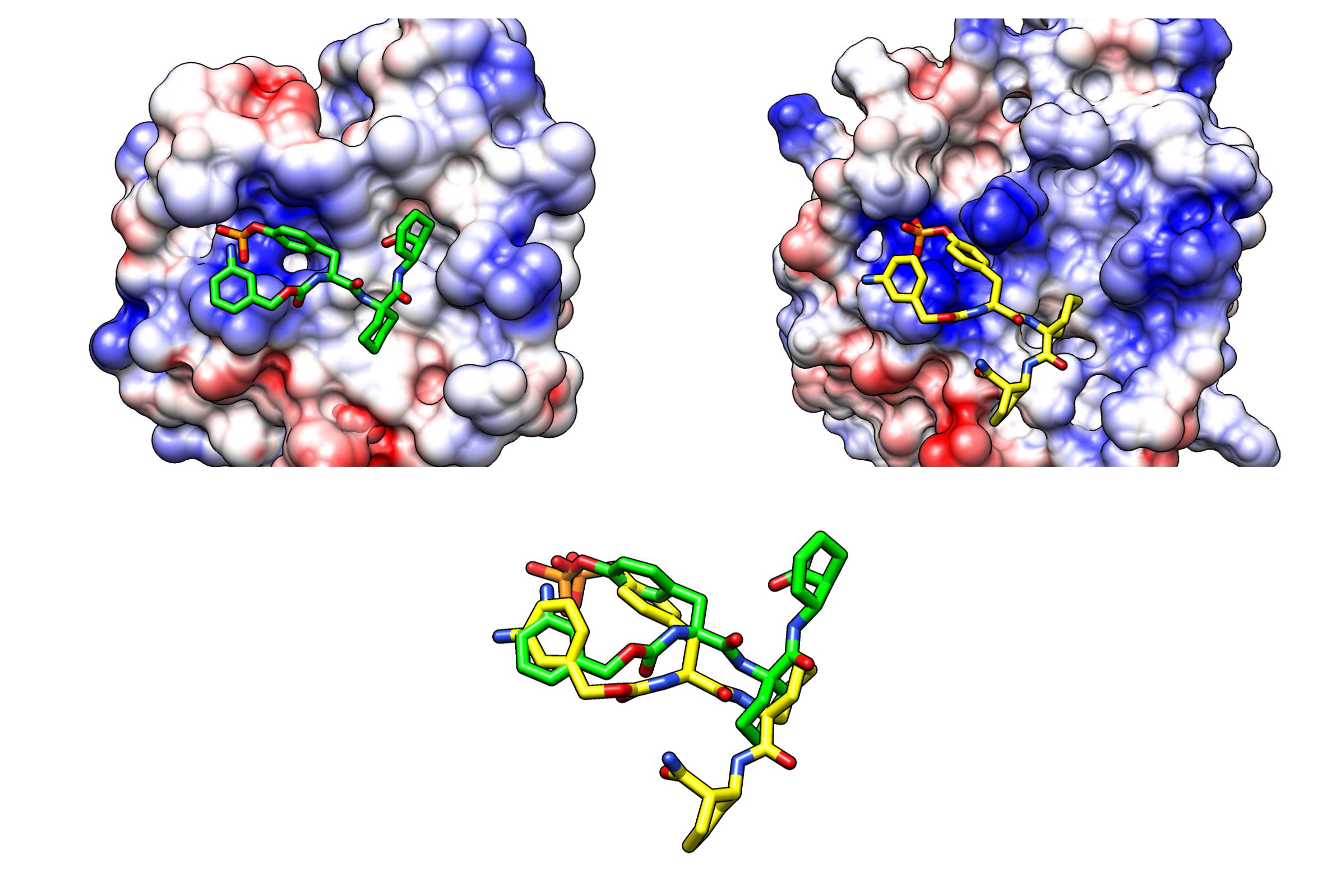

Supplement: Figure S14 — Protein-ligand complex with PDB ID 1CJ1. The experimental conformation (green) and the modeled conformation (yellow) of the ligand are shown in stick representation and the protein is shown in surface representation. (PNG) [file pone.0051603.s016.png]

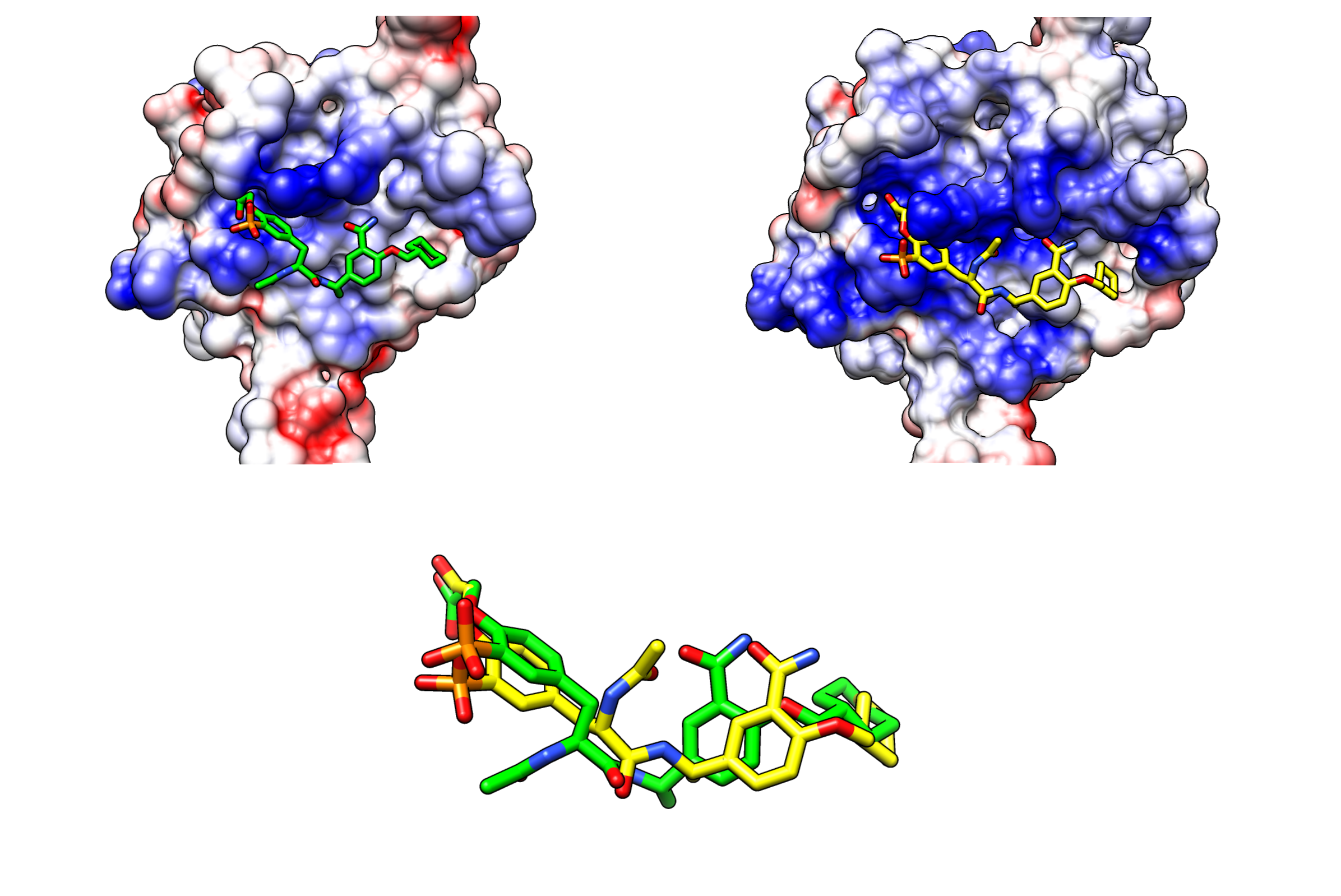

Supplement: Figure S15 — Protein-ligand complex with PDB ID 1IJR. The experimental conformation (green) and the modeled conformation (yellow) of the ligand are shown in stick representation and the protein is shown in surface representation. (PNG) [file pone.0051603.s017.png]

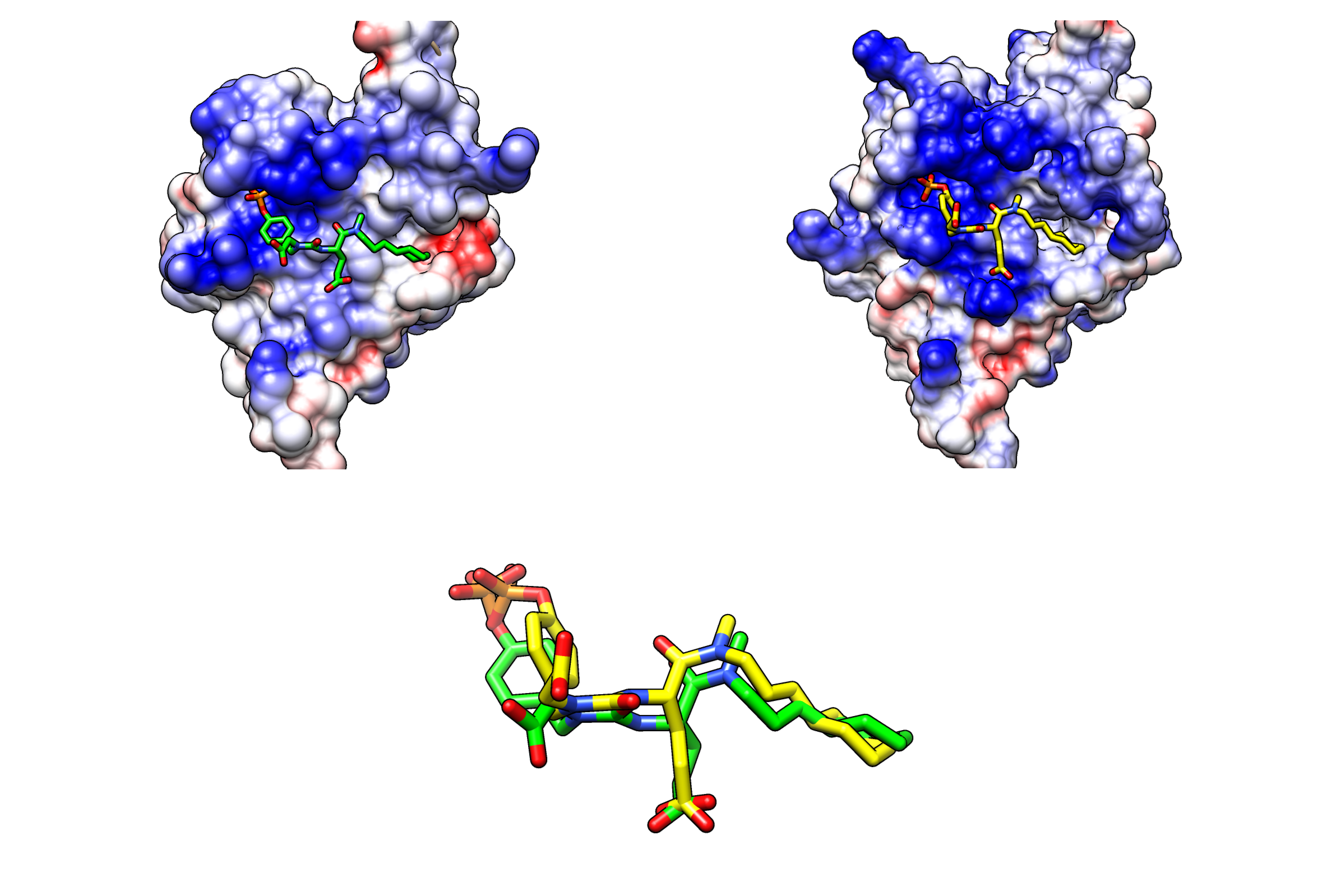

Supplement: Figure S16 — Protein-ligand complex with PDB ID 1SKJ. The experimental conformation (green) and the modeled conformation (yellow) of the ligand are shown in stick representation and the protein is shown in surface representation. (PNG) [file pone.0051603.s018.png]

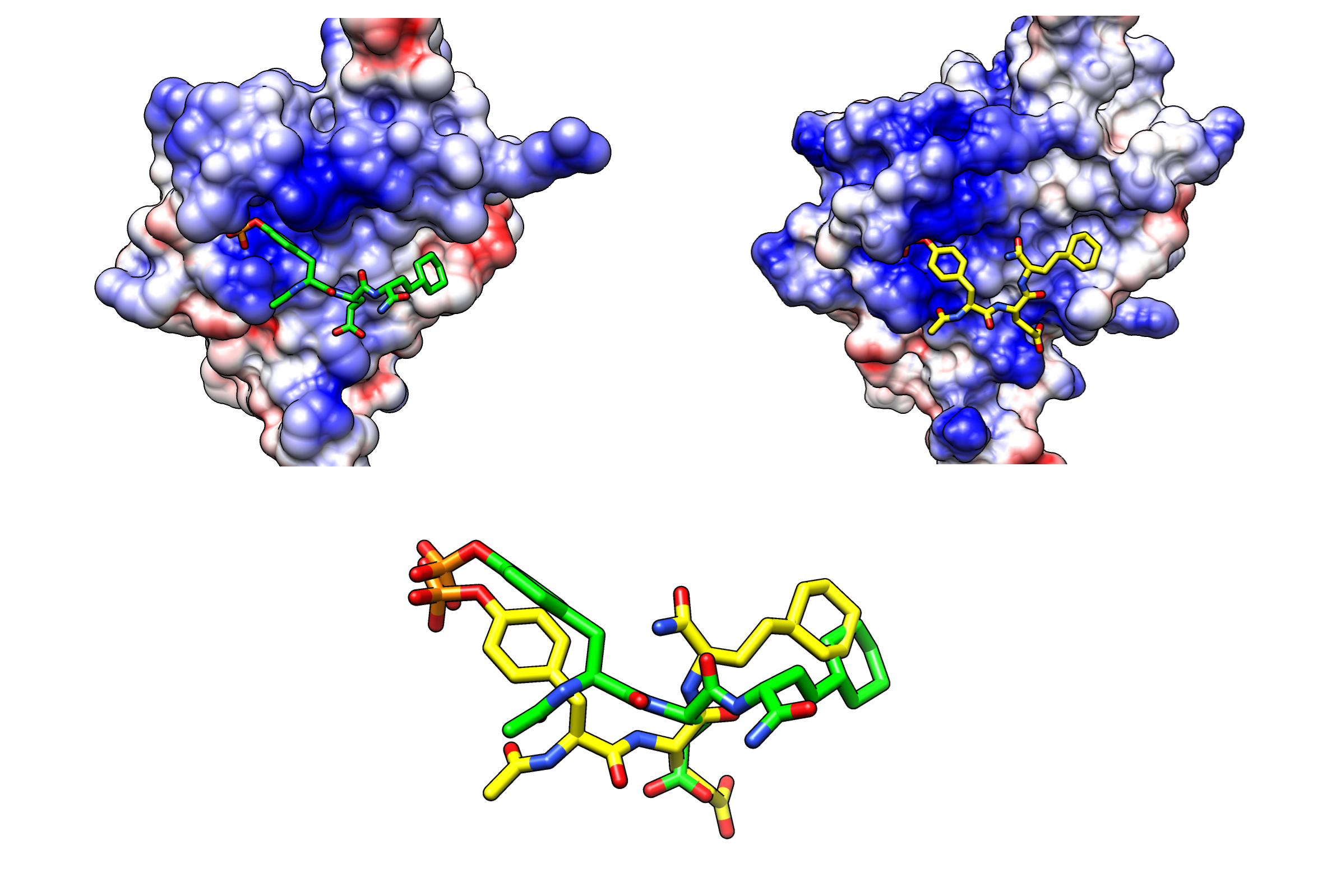

Supplement: Figure S17 — Protein-ligand complex with PDB ID 1BKM. The experimental conformation (green) and the modeled conformation (yellow) of the ligand are shown in stick representation and the protein is shown in surface representation. (PNG) [file pone.0051603.s019.png]

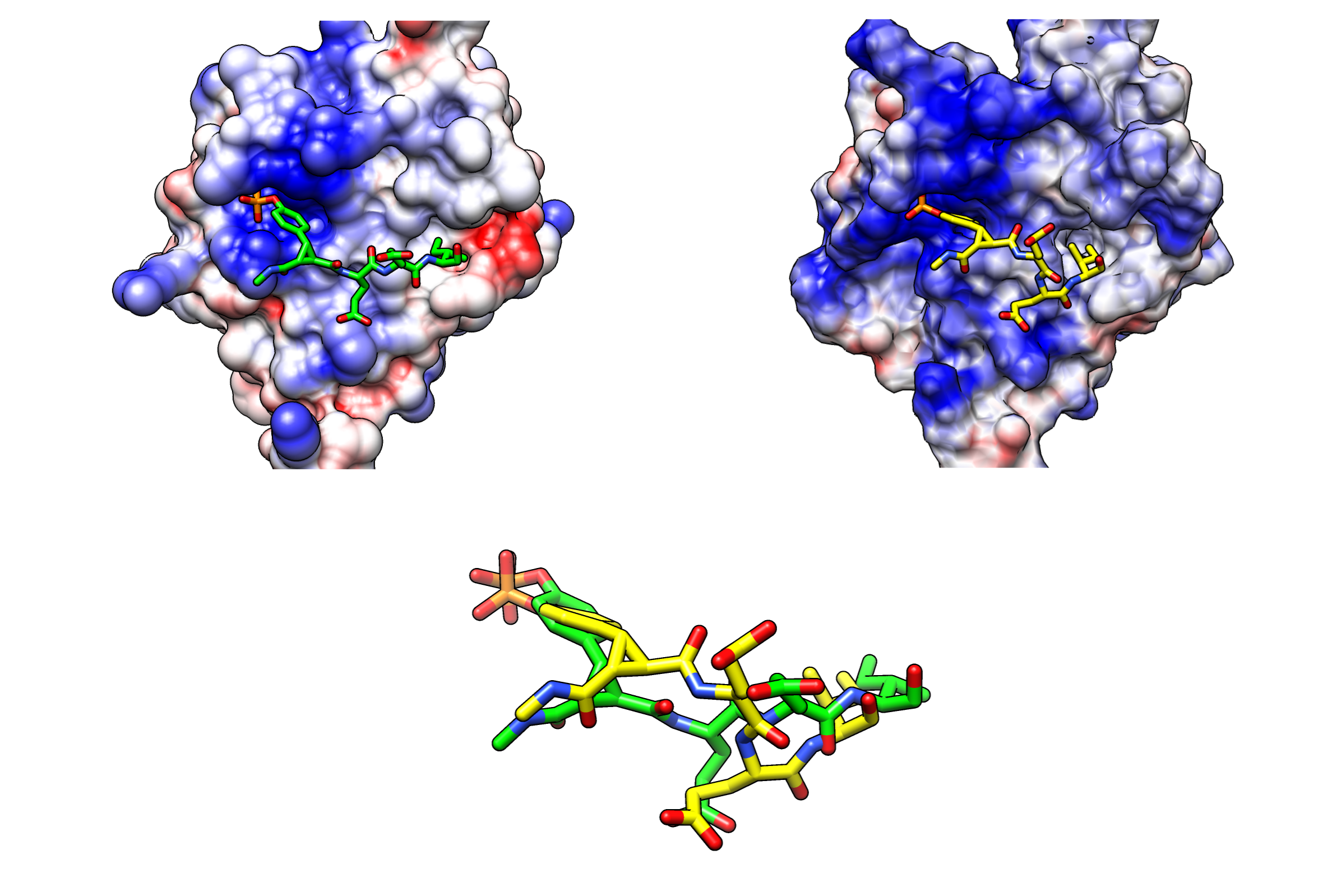

Supplement: Figure S18 — Protein-ligand complex with PDB ID 1IS0. The experimental conformation (green) and the modeled conformation (yellow) of the ligand are shown in stick representation and the protein is shown in surface representation. (PNG) [file pone.0051603.s020.png]

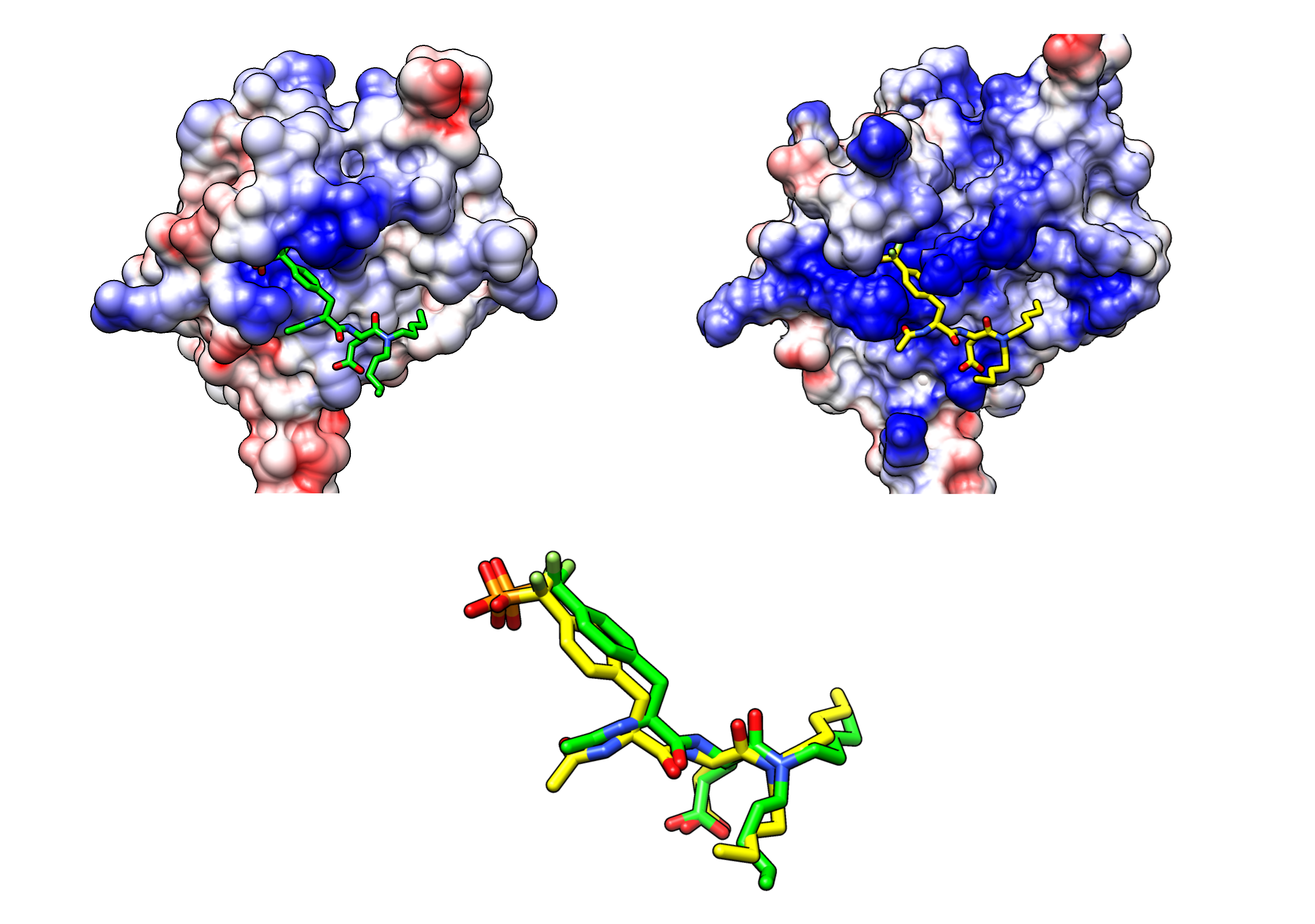

Supplement: Figure S19 — Protein-ligand complex with PDB ID 1A08. The experimental conformation (green) and the modeled conformation (yellow) of the ligand are shown in stick representation and the protein is shown in surface representation. (PNG) [file pone.0051603.s021.png]

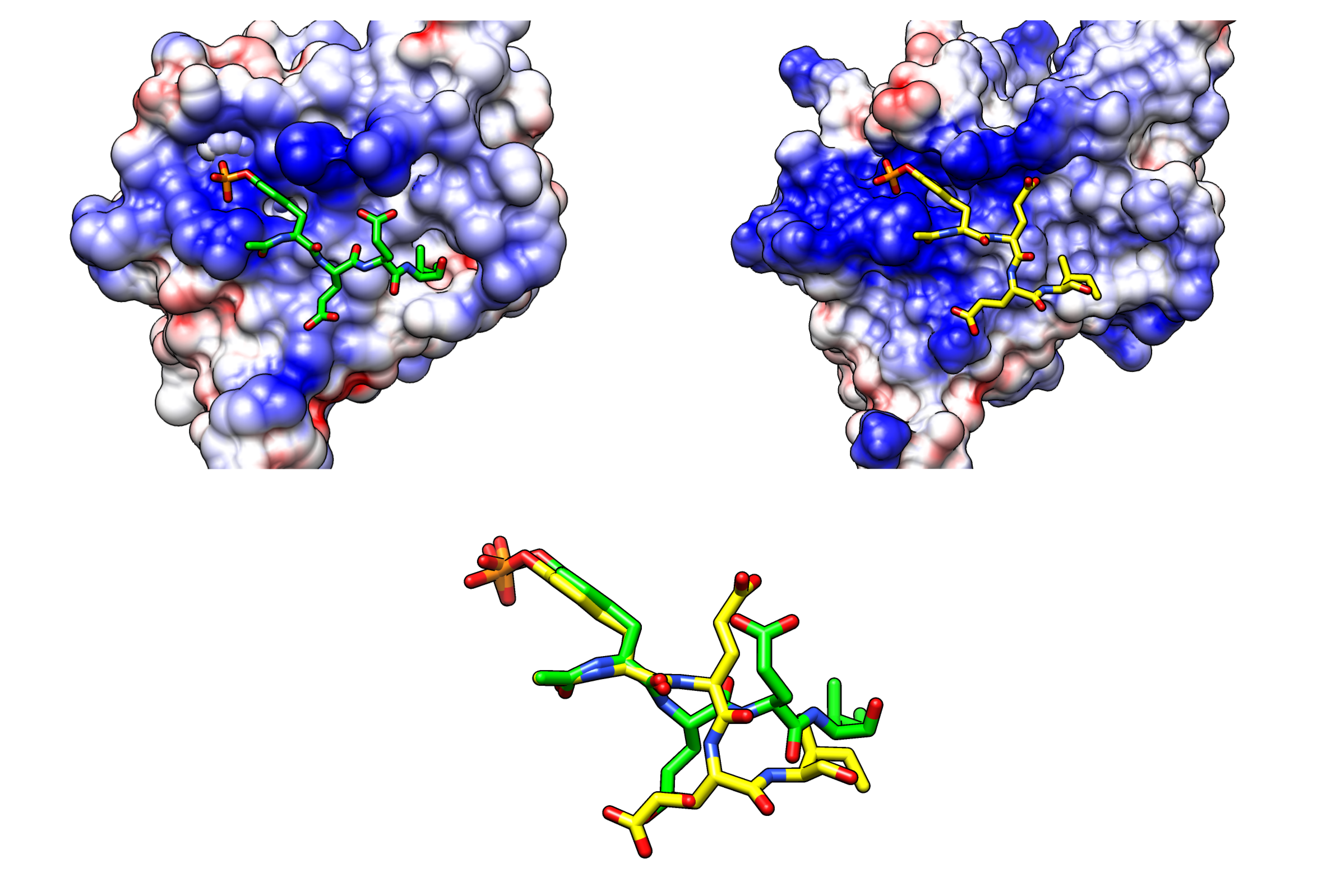

Supplement: Figure S20 — Protein-ligand complex with PDB ID 1SHD. The experimental conformation (green) and the modeled conformation (yellow) of the ligand are shown in stick representation and the protein is shown in surface representation. (PNG) [file pone.0051603.s022.png]

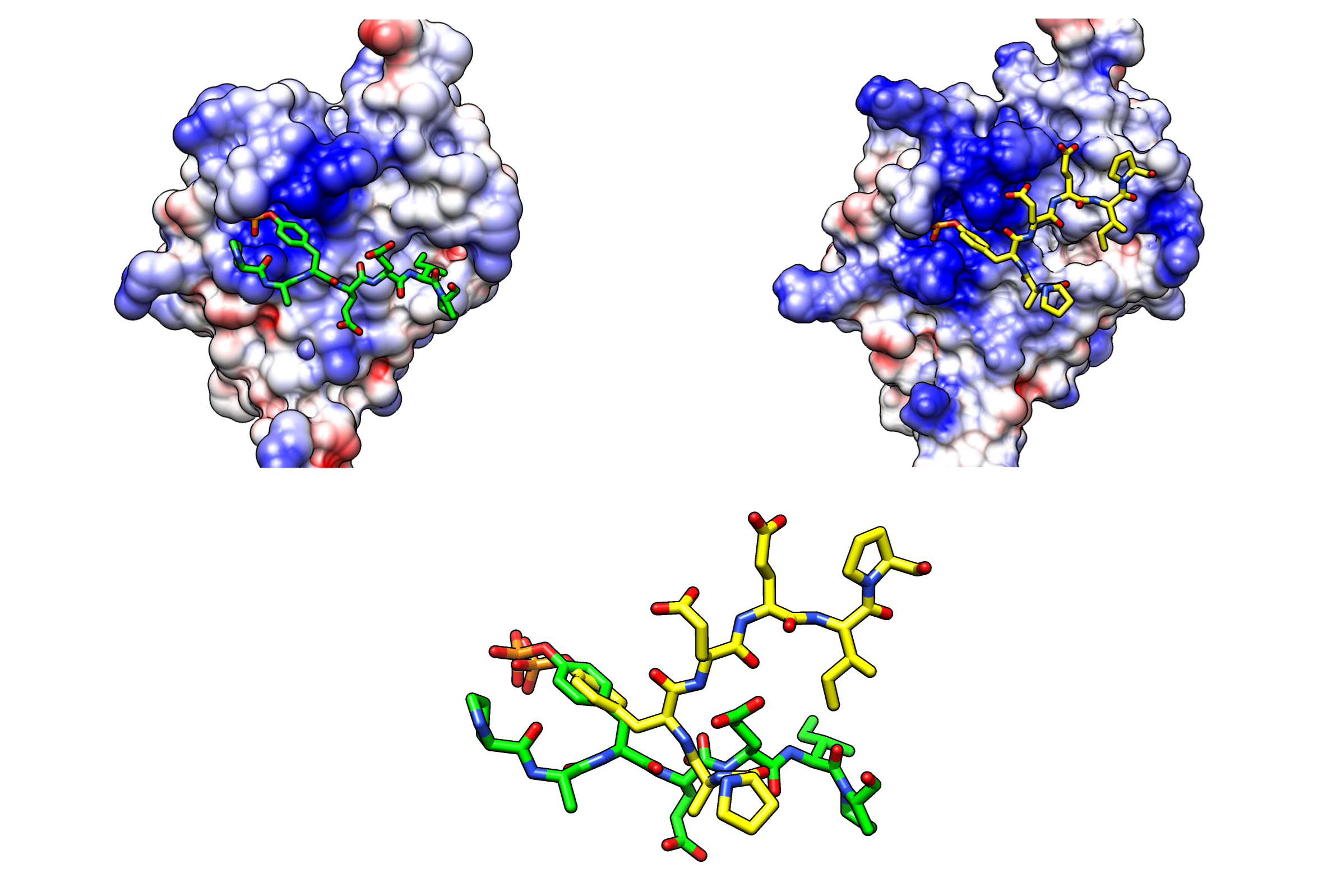

Supplement: Figure S21 — Protein-ligand complex with PDB ID 1SPS. The experimental conformation (green) and the modeled conformation (yellow) of the ligand are shown in stick representation and the protein is shown in surface representation. (PNG) [file pone.0051603.s023.png]

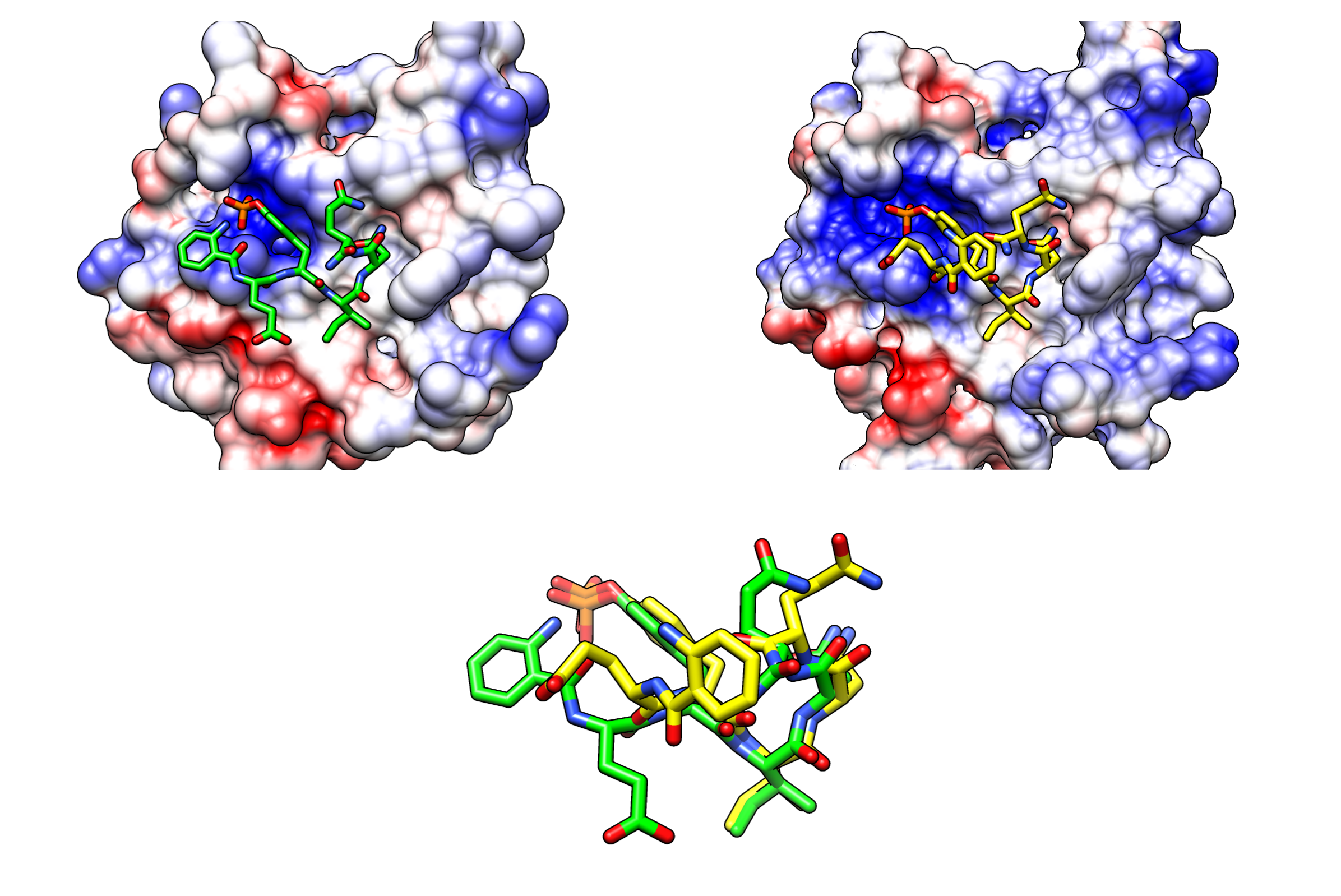

Supplement: Figure S22 — Protein-ligand complex with PDB ID 1ZFP. The experimental conformation (green) and the modeled conformation (yellow) of the ligand are shown in stick representation and the protein is shown in surface representation. (PNG) [file pone.0051603.s024.png]

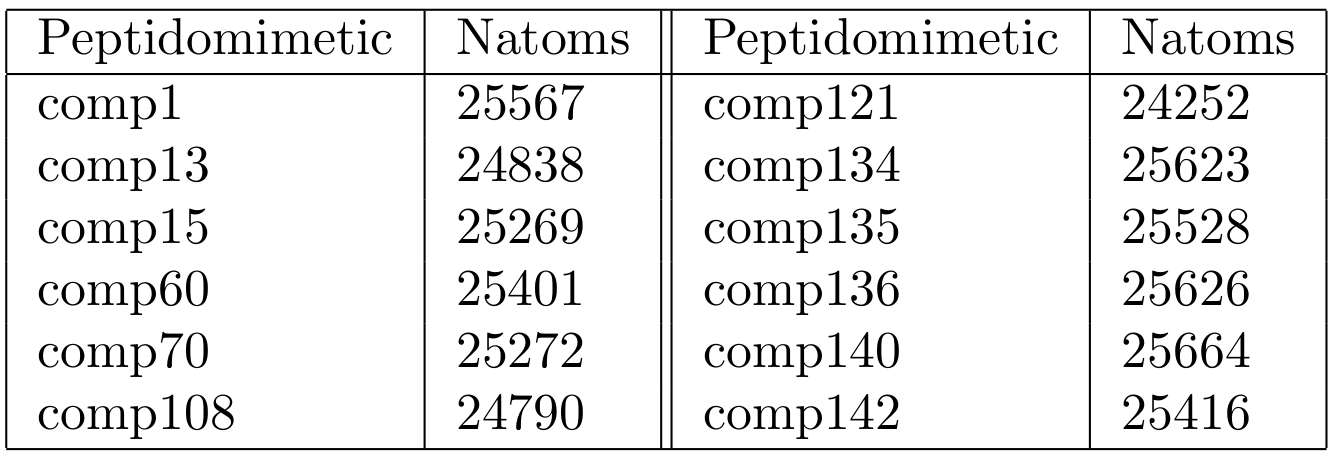

Supplement: Table S1 — Number of atoms in MD simulation systems. MD simulations were performed on 12 systems each comprising of one of the 12 peptidomimetics in complex with the SH2 domain of STAT3 in a explicit solvent box. This table lists the number of atoms (Natoms) in each system. (PNG) [file pone.0051603.s025.png]

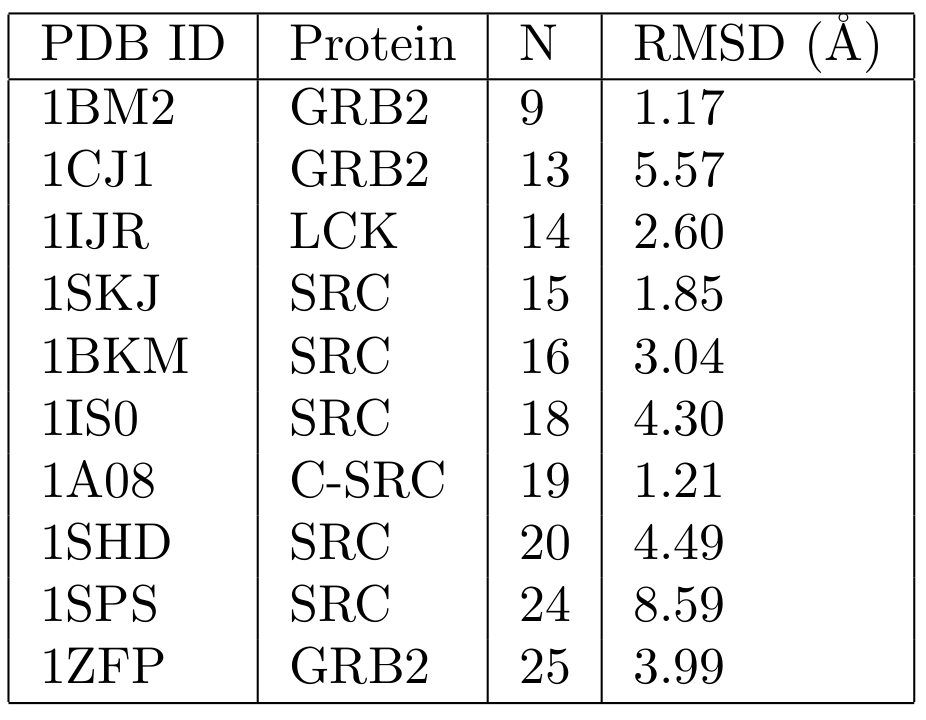

Supplement: Table S2 — Validation accuracy. 10 protein-ligand complexes were identified for validation of our modeling approach to predict binding modes of peptidomimetics in complex with the SH2 domain of STAT3. This table lists the PDB IDs that correspond to the deposited experimental structures of the 10 complexes. The RMSD values between the modeled conformation and experimenal conformation of the ligands evaluate the accuracy of our modeling approach. N represents the number of rotatable bonds in the ligands. (PNG) [file pone.0051603.s026.png]
